# A new type of sulfation reaction: *C*-sulfonation for α,β-unsaturated carbonyl groups by a novel sulfotransferase SULT7A1

**DOI:** 10.1093/pnasnexus/pgae097

**Published:** 2024-03-04

**Authors:** Katsuhisa Kurogi, Yoichi Sakakibara, Takuyu Hashiguchi, Yoshimitsu Kakuta, Miho Kanekiyo, Takamasa Teramoto, Tsuyoshi Fukushima, Takeshi Bamba, Jin Matsumoto, Eiichiro Fukusaki, Hiroaki Kataoka, Masahito Suiko

**Affiliations:** Department of Biochemistry and Applied Biosciences, Faculty of Agriculture, University of Miyazaki, Miyazaki 889-2192, Japan; Department of Biochemistry and Applied Biosciences, Faculty of Agriculture, University of Miyazaki, Miyazaki 889-2192, Japan; Department of Biochemistry and Applied Biosciences, Faculty of Agriculture, University of Miyazaki, Miyazaki 889-2192, Japan; Department of Bioscience and Biotechnology, Faculty of Agriculture, Kyushu University, Fukuoka 819-0395, Japan; Department of Bioscience and Biotechnology, Faculty of Agriculture, Kyushu University, Fukuoka 819-0395, Japan; Department of Bioscience and Biotechnology, Faculty of Agriculture, Kyushu University, Fukuoka 819-0395, Japan; Department of Pathology, Faculty of Medicine, University of Miyazaki, Miyazaki 889-1692, Japan; Division of Metabolomics, Medical Institute of Bioregulation, Kyushu University, Fukuoka 812-8582, Japan; Department of Applied Chemistry, Faculty of Engineering, University of Miyazaki, Miyazaki 889-2192, Japan; Department of Biotechnology, Graduate School of Engineering, Osaka University, Suita 565-0871, Japan; Department of Pathology, Faculty of Medicine, University of Miyazaki, Miyazaki 889-1692, Japan; Department of Biochemistry and Applied Biosciences, Faculty of Agriculture, University of Miyazaki, Miyazaki 889-2192, Japan

**Keywords:** sulfonation, sulfotransferase, SULT, α,β-unsaturated carbonyl, prostaglandin

## Abstract

Cytosolic sulfotransferases (SULTs) are cytosolic enzymes that catalyze the transfer of sulfonate group to key endogenous compounds, altering the physiological functions of their substrates. SULT enzymes catalyze the *O*-sulfonation of hydroxy groups or *N*-sulfonation of amino groups of substrate compounds. In this study, we report the discovery of *C*-sulfonation of α,β-unsaturated carbonyl groups mediated by a new SULT enzyme, SULT7A1, and human SULT1C4. Enzymatic assays revealed that SULT7A1 is capable of transferring the sulfonate group from 3′-phosphoadenosine 5′-phosphosulfate to the α-carbon of α,β-unsaturated carbonyl-containing compounds, including cyclopentenone prostaglandins as representative endogenous substrates. Structural analyses of SULT7A1 suggest that the *C*-sulfonation reaction is catalyzed by a novel mechanism mediated by His and Cys residues in the active site. Ligand-activity assays demonstrated that sulfonated 15-deoxy prostaglandin J_2_ exhibits antagonist activity against the prostaglandin receptor EP2 and the prostacyclin receptor IP. Modification of α,β-unsaturated carbonyl groups via the new prostaglandin-sulfonating enzyme, SULT7A1, may regulate the physiological function of prostaglandins in the gut. Discovery of *C*-sulfonation of α,β-unsaturated carbonyl groups will broaden the spectrum of potential substrates and physiological functions of SULTs.

**Significance Statement**

This study reports a new type of sulfation reaction, *C*-sulfonation, targeting α,β-unsaturated carbonyl group-containing compounds, such as cyclopentenone prostaglandins, and the catalyzing enzymes. Furthermore, the finding of antagonist activity of the sulfonated metabolites on prostaglandin receptors suggests a new mechanism for the regulation of prostaglandin function. The *C*-sulfonation of α,β-unsaturated carbonyl groups may serve as a defense system against oxidative stress, providing a new perspective on the physiological involvement of SULT-mediated sulfonation in a wide range of organisms, including humans.

## Introduction

**Fig. 1.**
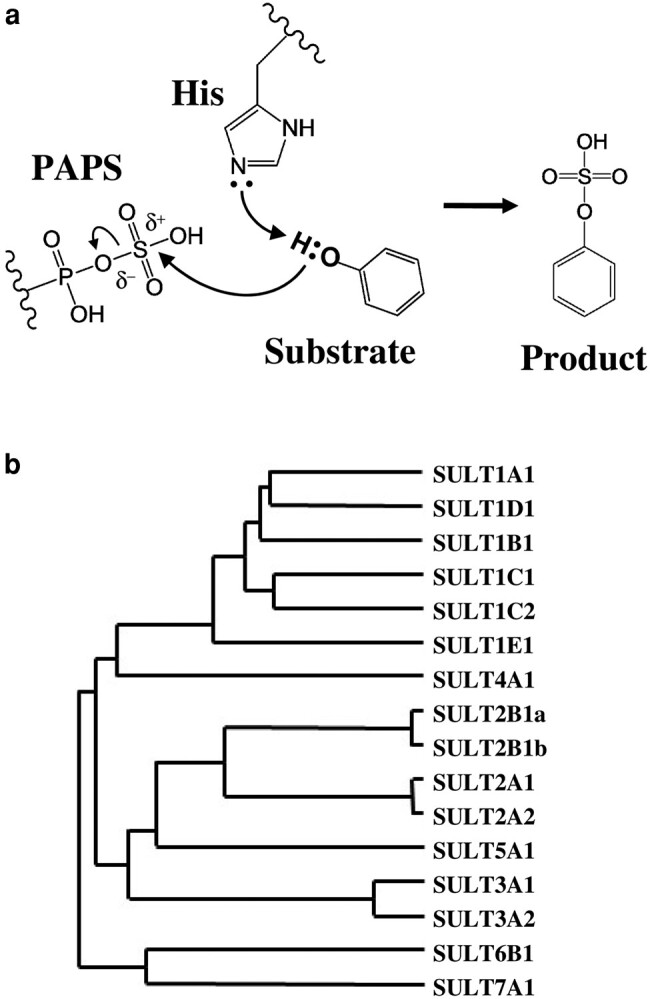
General sulfonation reaction mechanism and the classification of the mouse SULT7A1. a) Proposed reaction mechanism for the general sulfonation reaction catalyzed by SULTs. The phosphosulfate is a component of sulfonate donor, PAPS. b) Classification of a newly identified mouse SULT7A1 on the basis of deduced amino acid sequence. The dendrogram shows the degree of amino acid sequence homology among different mouse SULTs. Members sharing at least 45% amino acid sequence identity are classified into the same SULT gene family. For references of individual SULTs shown, refer to the review by Blanchard et al. (23).

On a par with the immune system, which functions to fend off bacteria and viruses invading the body, cytosolic sulfotransferase (SULT)-mediated sulfonation, in conjunction with other phase I and phase II detoxification reactions, serves to thwart the constant bombardment of numerous xenobiotics on living cells (1–3). In addition to its role in the “chemical defense” of living cells, sulfonation, as mediated by SULTs, plays a key role in maintaining the homeostasis of endogenous compounds, such as catecholamine hormones/neurotransmitters and thyroid/steroid hormones (4, 5). Sulfonated metabolites of endogenous compounds, such as dopamine sulfate and cholesterol sulfate, have been emerging as potential biomarkers for disorders in hormone metabolism and other pathophysiological conditions (6, 7). Thus, elucidating the physiological role of SULT-mediated sulfonation is an increasingly important issue. To date, a number of SULT members has been identified in a wide range of organisms, including bacteria, plants, and animals (8–11). Every reported SULT has been shown to catalyze the sulfonation of substrate compounds that contain a hydroxy or an amino group (9–11). An S_N_2-like in-line displacement mechanism (Fig. 1a) has been proposed for SULT-mediated reactions involving the transfer of a sulfonate group (–SO_3_H) from 3′-phosphoadenosine 5′-phosphosulfate (PAPS) to a hydroxy group of the substrate (12, 13). By confining to this widely accepted viewpoint, compounds that may undergo sulfonation at locations other than the hydroxy or amino group thus may be overlooked. Indeed, a recent study reported the *O*-sulfonation of ketosteroids, having neither hydroxy nor amino groups, by a hydroxysteroid SULT, SULT2A1 (15). The discovery of ketosteroid *O*-sulfonation clearly suggests that SULT enzymes may be capable of modifying not only hydroxy and amino groups but also carbonyl groups. Thus, the basic concept regarding the targeting groups of substrate compounds has lost sight of the potential substrates for SULTs, including orphan SULTs, and their physiological functions.

α,β-Unsaturated carbonyl groups contain a highly reactive electrophilic β-carbon where Michael addition occurs (16). A variety of α,β-unsaturated carbonyl compounds are produced *in vivo* by lipid peroxidation, leading to various physiological effects (16, 17). Cyclopentenone prostaglandins (PGA_2_ and 15-deoxy-D^12,14^-PGJ_2_ (15d-PGJ_2_)) exert the apoptotic and anti-inflammatory effects on cancer cells through the α,β-unsaturated carbonyl group (18). Dopamine oxidation, leading to the production of dopamine quinone, has also been reported to contribute to the neurodegenerative diseases through cytotoxicity (19). In addition, xenobiotic quinones in air pollutants can lead to bronchial disorders by covalently binding to the cysteine group of GSH and proteins (20, 21). The antipyretic agent acetaminophen has been reported to be metabolized into a reactive metabolite, *N*-acetyl-*p*-benzoquinone imine, in case of overdose, leading to hepatotoxicity (22). Therefore, metabolism of α,β-unsaturated carbonyl compounds plays an important role in regulation of the positive health effects and detoxification of the cytotoxic effects. α,β-Unsaturated carbonyl compounds are mainly metabolized through β-oxidation and glutathione conjugation (14, 15). Although glutathione conjugation attenuates the reactivity and toxicity of α,β-unsaturated carbonyl compounds (14), an intensive glutathione conjugation reaction can lead to intracellular GSH depletion. Other phase II reactions, including glucuronidation and sulfonation, for these compounds have not been reported.

To date, a total of six SULT families have been identified and well studied in vertebrate animals, including mice and humans. This study addresses the characterization of a newly identified SULT (annotated as Sult6B2 or Sult6B1-like in database), ruling out its classification into the SULT7 family based on the established SULT nomenclature system, and specifically focuses on *C*-sulfonation of α,β-unsaturated carbonyl groups mediated by SULT7A1. Molecular cloning, expression, and purification were first performed to prepare the new mouse SULT7A1 enzymes. Enzyme assay with simple key component compounds was used to identify a core structure, α,β-unsaturated carbonyl group, of potential substrates for SULT7A1, leading to the identification of cyclopentenone prostaglandins as its physiological substrates. Further investigations were carried out to explore the *C*-sulfonation of cyclopentenone prostaglandin occurring at α,β-unsaturated carbonyl group through structural, expression, ligand-activity analyses as well as enzymatic approaches.

## Results

### Identification of a new SULT enzyme, SULT7A1

A novel *SULT* gene (gene ID: 330440), annotated as *Sult6B2* in January 2024, was identified in mouse genome database (NCBI) and performed molecular cloning of this novel mouse SULT. The full-length nucleotide sequence obtained from the results of rapid amplification of cDNA ends (RACE) showed that the novel *SULT* gene generates an mRNA containing a 150-bp 5′-UTR sequence, an open-reading frame (ORF) of 873 nucleotides encoding 290 amino acid polypeptides and a 750-bp 3′-UTR (Fig. S1). Sequence analysis revealed that this novel mouse SULT displays 29.7, 27.7, 29.7, 24.7, 26.9, and 32.4% amino acid sequence identity to mouse SULT1A1, SULT2A1, SULT3A1, SULT4A1, SULT5A1, and SULT6B1, respectively. Since members of the same *SULT* gene family generally share at least 45% amino acid sequence identity (23), the newly cloned mouse SULT was judged to belong to a novel SULT family, SULT7, and designated SULT7A1 rather than SULT6B2, as annotated in the NCBI database (Fig. 1b). Analysis of the amino acid sequence of this novel SULT7A1 showed that it contains a conserved catalytic residue His^94^ and the signature sequences, called the 5′-phosphosulfate-binding loop, 3′-phosphate-binding loop, and p-loop, that are responsible for the binding to the sulfonate group donor, PAPS (24, 25) (Figs. S1 and S2).

### Specific sulfonation of α,β-unsaturated carbonyl groups by SULT7A1

**Fig. 2.**
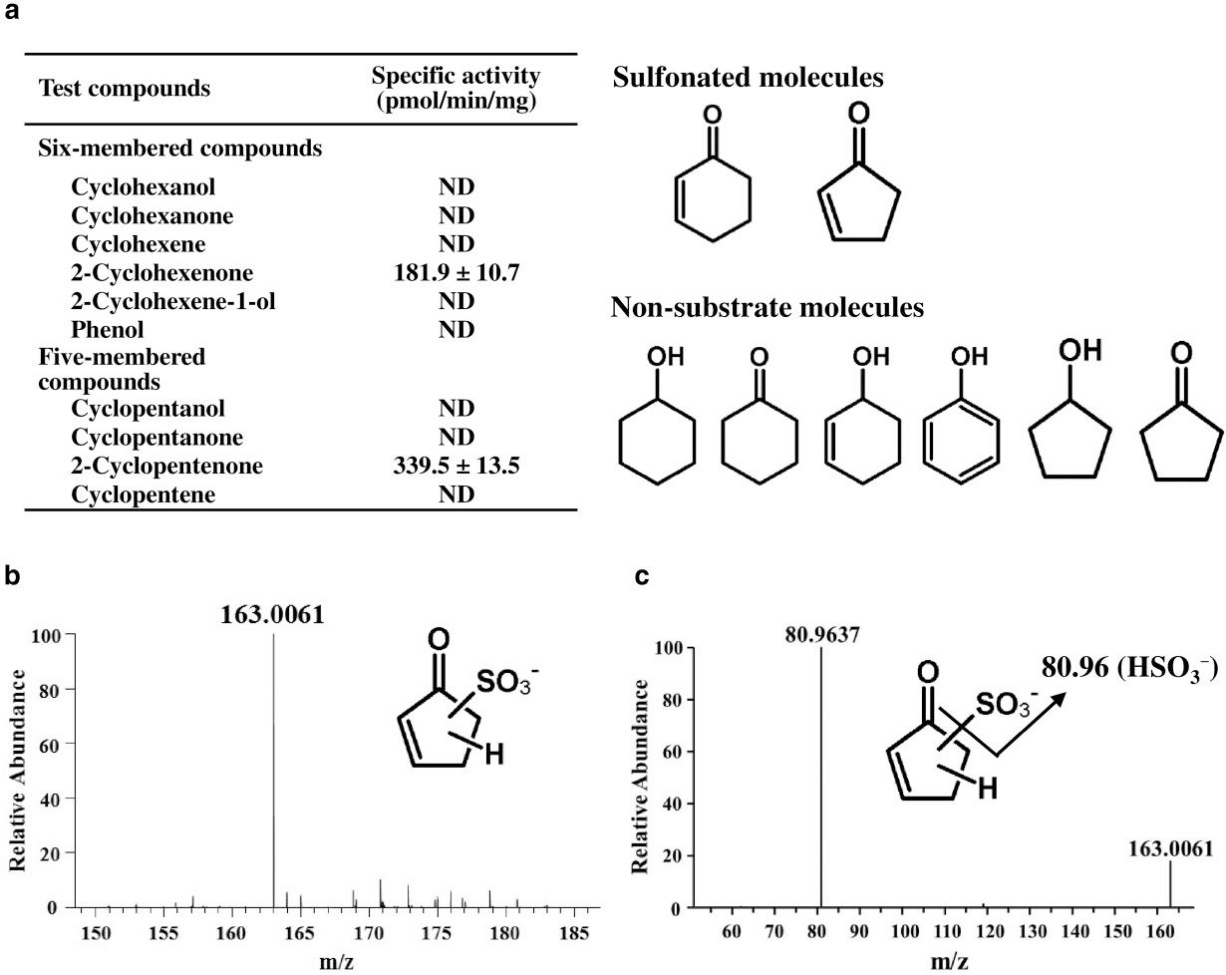
Unique sulfonating activity of SULT7A1 toward α,β-unsaturated carbonyl groups. a) Specific sulfonating activity of SULT7A1 toward five- or six-membered ring compounds (10 μM substrate concentration). The activity refers to pmol substrate sulfonated/min/mg purified enzyme. ND refers to activity not detected with the detection limit estimated to be 1.0 pmol/min/mg protein. Data represent the mean ± SD derived from three determinations. b) Full mass spectrum of sulfonated derivatives of 2-cyclopentenone. The eluted fraction with 1% NH_4_OH in methanol was subjected to direct-infusion MS in a full mass scan mode. c) MS/MS spectrum of 163.0061 *m*/*z*.

Purified recombinant SULT7A1 was subjected to the sodium dodecylsulfate–polyacrylamide gel electrophoresis (SDS–PAGE). Recombinant SULT7A1 migrated as a single 33.4-kDa protein band as calculated (Fig. S3). Sulfotransferase assays were carried out under the conditions described in the Materials and methods section. Among a variety of simple six-membered ring compounds tested as substrates, SULT7A1 showed a specific sulfonating activity toward 2-cyclohexenone, while exhibiting no activity toward other compounds, such as cyclohexanone and 2-cyclohexene-1-ol, at concentrations ranging from 1 to 1,000 μM as well as phenol (Fig. 2a). The enzyme-mediated reaction was also confirmed using the negative control in the presence of enzymes heated at 98 °C for 3 min. Five-membered ring-containing compounds were also tested, and interestingly, SULT7A1 showed sulfonating activity only toward 2-cyclopentenone (Fig. 2a). These results clearly indicated that substrates to be sulfonated by SULT7A1 should contain an α,β-unsaturated carbonyl group. Kinetic studies showed that SULT7A1 exhibited the Michaelis–Menten kinetics for the sulfonation of 2-cyclohexenone and 2-cyclopentenone with 16.4 and 40.6 μM of *K*_m_ values, respectively (Fig. S4 and Table 1). On the other hand, SULT7A1 showed the low *K*_m_ value (0.2 μM) with PAPS. Therefore, SULT7A1 exhibits the higher affinity toward 2-cyclohexenone than 2-cyclopentenone with the higher affinity toward PAPS.

**Table 1.** Kinetic constants of the sulfonation mediated by the mouse SULT7A1.^a^

	*V*_max_	*K*_m_	*V*_max_/*K*_m_
	(pmol/min/mg)	(μM)	(μL/min/mg)
2-Cyclopentenone	1,702 ± 97	40.61 ± 4.10	41.91
2-Cyclohexenone	485.5 ± 14.3	16.37 ± 1.19	29.66
PGA_2_	267.5 ± 22.8	186.1 ± 29.8	1.44
PGJ_2_	426.2 ± 21.6	64.71 ± 9.26	6.59
Δ^12^-PGJ_2_	478.1 ± 23.5	45.43 ± 5.43	10.52
15d-PGJ_2_	453.8 ± 18.6	66.20 ± 5.68	6.85
PAPS^b^	491.1 ± 15.1	0.20 ± 0.02	2,517

^a^Results represent the means ± SD derived from three determinations. Kinetic parameters were determined based on the equation for Michaelis–Menten kinetics. ^b^10 μM of 2-cyclopentenone was used for a sulfonate-acceptor substrate.

### Mass spectrometry analysis of the sulfonated reaction product of 2-cyclopentenone

To validate the specificity of SULT7A1 for α,β-unsaturated carbonyl-containing compounds, the sulfonated product of 2-cyclopentenone was analyzed using mass spectrometry (MS). A prominent ion peak at *m*/*z* of 163.0061 was detected for the sulfonated product isolated from SULT7A1-mediated 2-cyclopentenone sulfonation reaction mixture (Fig. 2b). MS/MS analysis of the ion at *m*/*z* of 163.0061 generated a fragment ion at *m*/*z* of 80.9637, corresponding to a sulfonate ion, HSO_3_^−^ (Fig. 2c). Its ^34^S-isotopic ion at *m*/*z* of 165.0019 was also found with an intensity of 4.53% of the monoisotopic ion peak (163.0061 *m*/*z*; Fig. S5a). The isotopic ion peak further generated a ^34^S-isotopic sulfonate fragment ion, *m*/*z* of 82.9595 (Fig. S5b), supporting that the peak with *m*/*z* of 163.0061, detected in Fig. 2b, carries a sulfur atom and corresponds a deprotonated ion, [C_5_H_8_O_4_S–H]^−^. The nominal mass number of the sulfonated product of 2-cyclopentenone increased by 82 from the substrate (C_5_H_6_O: molecular weight, 82) in the noncharged condition. These observations suggested that the sulfonation reaction of α,β-unsaturated carbonyl-containing 2-cyclopentenone indeed occurred, with the transfer of a sulfonate group (–SO_3_H: molecular weight, 81) plus one hydrogen atom.

### Sulfonation of cyclopentenone prostaglandins by SULT7A1

**Fig. 3.**
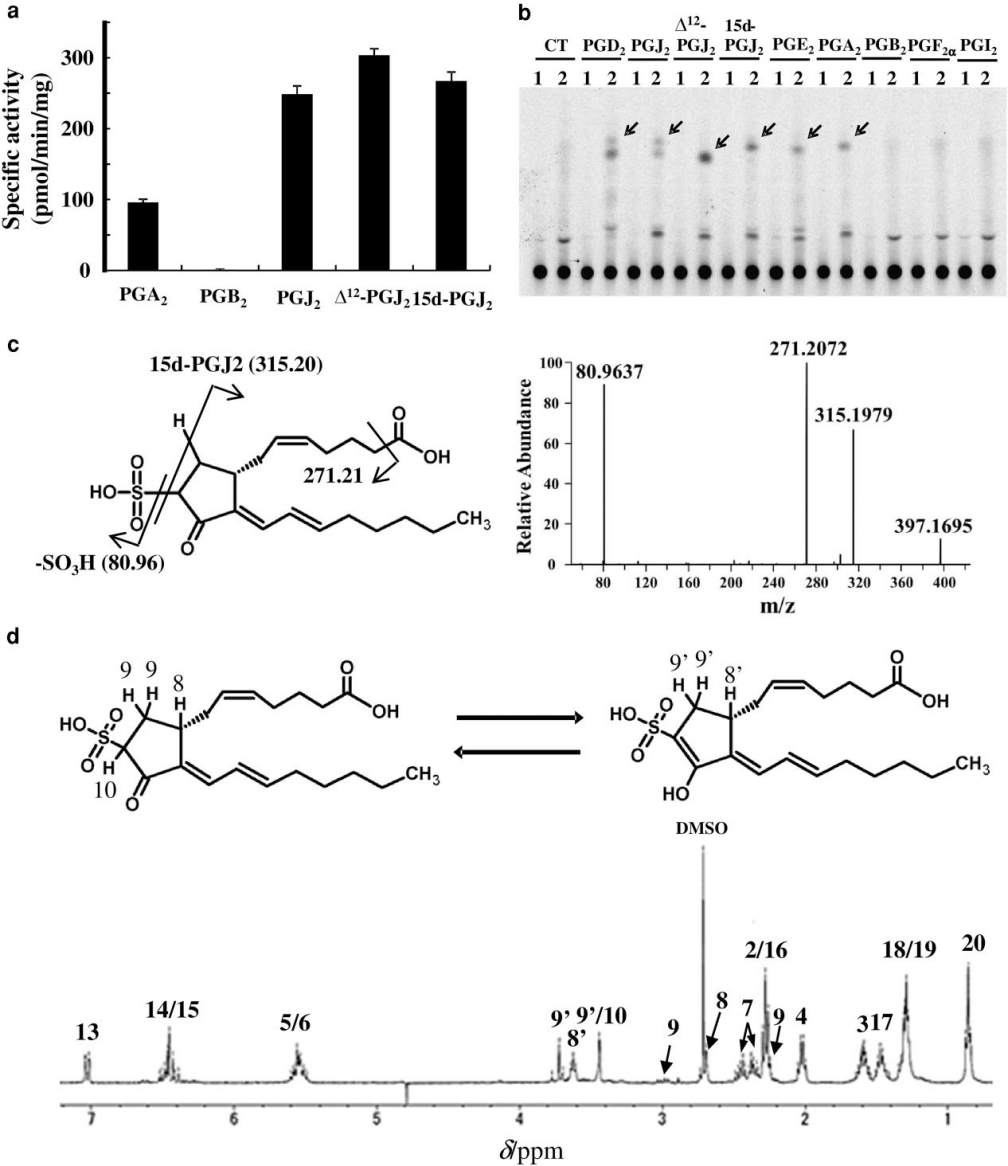
Sulfonation of cyclopentenone prostaglandins as mediated by SULT7A1. a) Specific sulfonating activity of SULT7A1 toward cyclopentenone prostaglandins (100 μM substrate concentration). Data represent the mean ± SD derived from three determinations. b) Analysis of [^35^S]sulfonated products of prostaglandins generated by SULT7A1-expressing BHK cells. The figure shows the autoradiograph taken from the TLC plate used for the analysis of spent labeling media. Lanes 1 and 2 correspond to the labeling media of cells transfected with pEF6 (lane 1) or pEF6-SULT7A1 (lane 2). CT (Control) refers to the control-labeling medium without added prostaglandins. The arrows indicate the sulfonated derivatives. c) MS/MS analysis of the sulfonated product of 15d-PGJ_2_. The MS/MS spectrum is obtained from a parent ion peak at 397.17 *m*/*z*. d) ^1^H-NMR analysis of the sulfated product of 15d-PGJ_2_. Each proton numbered in chemical structures is assigned for the corresponding spectrum.

In order to gain insight into the physiological functions of SULT7A1, cyclopentenone prostaglandins carrying a 2-cyclopentenone structure with an electrophilic β-carbon were further analyzed as representative endogenous α,β-unsaturated carbonyl-containing compounds. Five cyclopentenone prostaglandins, prostaglandin A_2_ (PGA_2_), PGB_2_, PGJ_2_, Δ^12^-PGJ_2_, 15-deoxy-Δ^12^, ^14^-PGJ_2_ (15d-PGJ_2_), were tested as substrates for SULT7A1. As shown in Fig. 3a, SULT7A1 displayed significant sulfonating activity toward all tested compounds except PGB_2_. The enzyme-mediated sulfonation of 15d-PGJ_2_ with unlabeled PAPS was also detected by HPLC at 301 nm (Fig. S6a). Kinetic studies showed that SULT7A1 exhibited the Michaelis–Menten kinetics for the sulfonation of all four cyclopentenone prostaglandins tested (Fig. S4). SULT7A1 showed the *K*_m_ values about 50 μM with PGJ_2_, Δ^12^-PGJ_2_, and 15d-PGJ_2_, while SULT7A1 showed the high *K*_m_ value with PGA_2_ (Table 1). Cyclopentenone prostaglandins are known to be generated from the spontaneous metabolism of PGD_2_ and PGE_2_ (26, 27). To investigate whether the sulfonation is indeed involved in the cellular prostaglandin metabolism, a metabolic labeling experiment was performed with baby hamster kidney (BHK-21) cells engineered to transiently express SULT7A1. Consistent with the enzymatic assay data, BHK-21 cells expressing SULT7A1 were found to generate sulfonated products derived from PGD_2_ and PGE_2_ as well as other four cyclopentenone prostaglandins, but not three other prostaglandins, PGB_2_, PGF_2α_, and PGI_2_ (Fig. 3b).

### Structure analysis of the sulfonated 15d-PGJ_2_

To clarify the chemical structure of sulfonated cyclopentenone prostaglandins, MS and NMR analyses were performed using 15d-PGJ_2_ as a representative cyclopentenone prostaglandin. MS/MS analysis of the ion at *m*/*z* of 397.1695 generated fragment ions at *m*/*z* of 315.1979 corresponding to 15d-PGJ_2_, [C_20_H_27_O_3_]^−^, and 80.9637 corresponding to a sulfonate ion, HSO_3_^−^ (Fig. 3c). Its ^34^S-isotopic ion at *m*/*z* of 399.1653 was found with an intensity of 4.57% of monoisotopic ion peak (397.1695 *m*/*z*; Fig. S6b). The isotopic ion peak further generated a ^34^S-isotopic sulfonate fragment ion, *m*/*z* of 82.9594 (Fig. S6c). Therefore, MS and MS/MS data of sulfonated 15d-PGJ_2_, with an *m*/*z* of 397.17, indicated the addition of a sulfonate group plus one hydrogen atom to 15d-PGJ_2_, which is consistent with the data for sulfonated 2-cyclopentenone (Figs. 2bc and S5). ^1^H-NMR data including ^1^H–^1^H COSY showed that the unsaturated proton peaks for H9 (7.66 ppm) and H10 (6.32 ppm) of 15d-PGJ_2_ were shifted or disappeared, suggesting that the sulfonation reaction likely produced two different products with the sulfonate group located at C10 (Figs. 3d and S7, and Table S1). The major product appeared to be in the enol form, likely converted from the other product in the keto form. The transfer of the sulfonate group to a carbon atom suggested that SULT7A1 catalyzes an unprecedented sulfonation reaction with a distinct reaction mechanism.

### Unique structure of SULT7A1 and the reaction mechanism of the *C*-sulfonation of α,β-unsaturated carbonyl groups

**Fig. 4.**
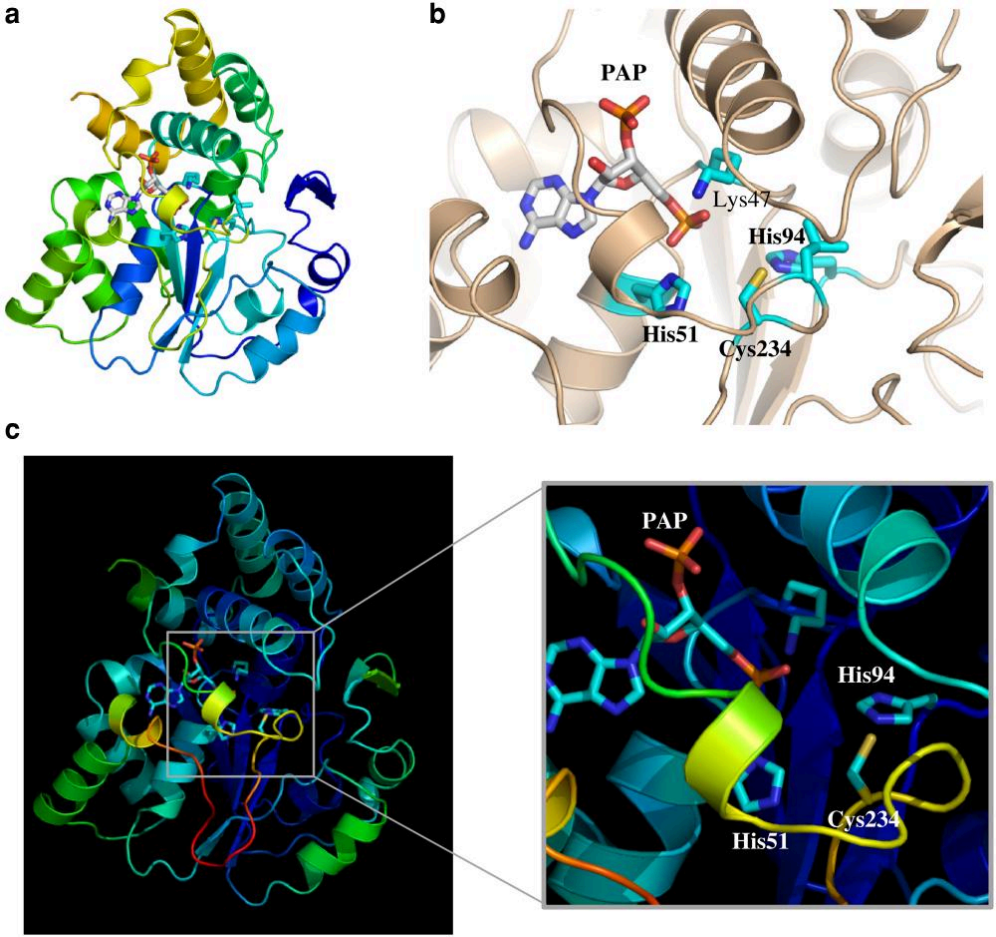
Crystal structure of SULT7A1 in complex with PAP. a) Ribbon diagram of the overall structure of SULT7A1 in complex with PAP. b) Close-up view of the active site of SULT7A1 structure. The stereo view of the PAP-docking site is shown. Potential amino acid residues around the sulfate portion of PAPS are colored slate blue. c) Crystallographic B-factors of the overall structure of SULT7A1. B-factor data are displayed with a color code from blue to red, indicating an increase in B-factor numbers. The right panel shows a close-up view of the active site of SULT7A1.

**Fig. 5.**
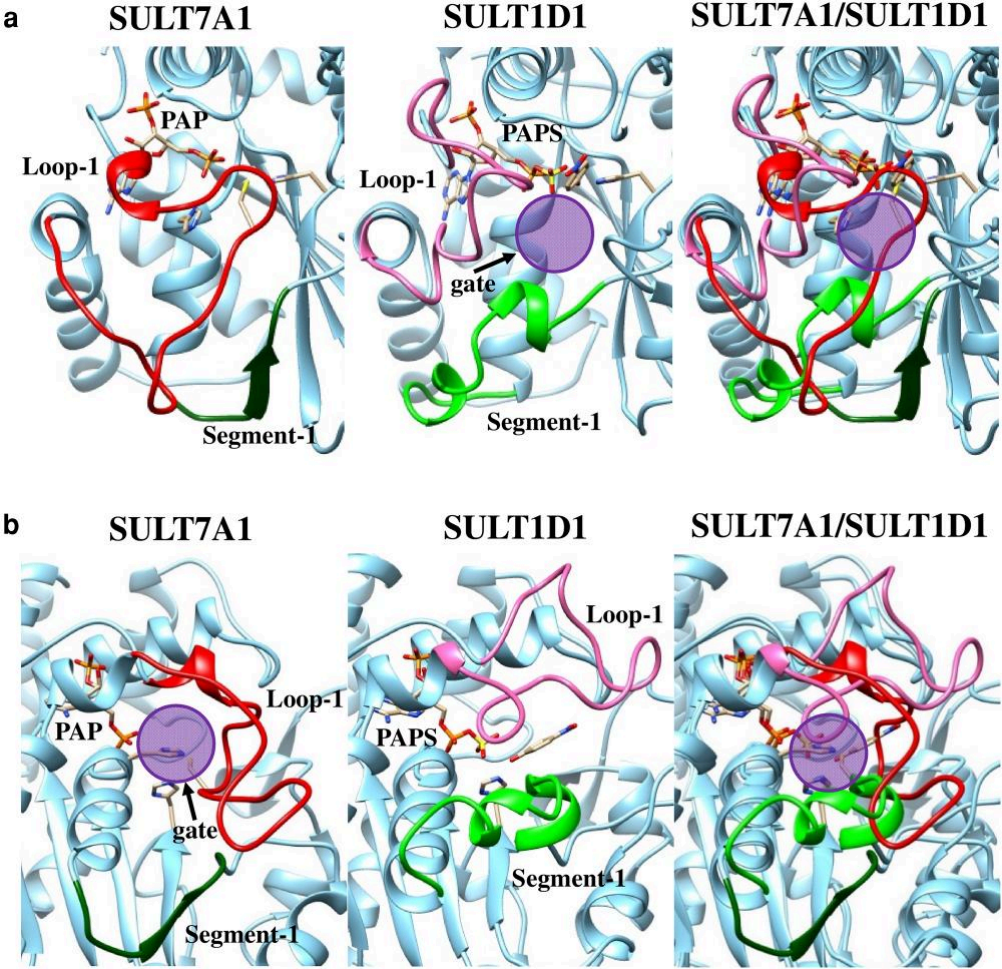
Putative substrate entry gate of SULT7A1 in comparison with SULT1D1. A close-up view of the substrate entry gate of SULT1D1 and SULT7A1 is aligned in the right panel. The ribbon diagram of loop-1 and alternative segment-1 is colored in red and dark green for SULT7A1 (left panel) and pink and green for SULT1D1 (middle panel). The substrate entry gates for SULT1D1 (a) and SULT7A1 (b) were colored in purple. The alignment of SULT1D1 (Protein Data Bank code: 2ZVP) and SULT7A1 was performed under the Needleman–Wunsch algorithm with BLOSUM62 matrix implemented in the USCF Chimera software (30, 31). The substrate entry gate of SULT7A1 was proposed based on the results of deletion of Cys^234^.

To probe further the mechanism underlying this unprecedented *C*-sulfonation reaction, structural and mutational analyses were performed. Although the overall structure of SULT7A1 with 3′-phosphoadenosine-5′-phosphate (PAP) appeared relatively similar to that of mouse SULT1D1, which catalyzes primarily the sulfonation of phenolic compounds (28, 29), a unique hydrogen bond formed between His^94^ and Cys^234^ is present in the active site (Figs. 4a, b and S8a). It is noted that the Cys^234^-containing loop (loop-1) occupies the space for a substrate compound docked into the active site (Fig. S8b). The Cys^234^-containing loop, however, is flexible based on the high B-factor number (Fig. 4c). Although loop-1 and segment-1 of SULT1D1 are the key regions forming the substrate entry gate, the flexible loop-1 of SULT7A1 disrupts the entry gate conserved in the other SULTs (Fig. 5a). Substrate entry gate was not observed in the SULT7A1 structure, but the flexible loop-1 and α-helix following the alternative segment-1 of SULT7A1 may form a potential substrate entry gate (Fig. 5b). Deleting Cys^234^ residue appeared to open the substrate entry gate, connecting to active site, formed by the flexible loop-1 (Fig. S9a). The flexible loop-1 may, therefore, play a pivotal role in the acceptance of a substrate and catalytic action. Indeed, the C234A mutated SULT7A1 failed to display sulfonating activity toward 2-cylohexenone and 2-cyclopentenone over a wide range of concentrations (1–1,000 μM) tested. In addition to C234A, H51A and H94A also showed no activity to the substrates tested. These findings imply that upon docking of a substrate, the conformation of Cys^234^-containig loop may be altered and the substrate reaching the active site may then disrupt the hydrogen bound between Cys^234^ and His^94^, thereby gaining access to the co-substrate PAPS. It, therefore, appears that His^51^, His^94^, and Cys^234^ are essential residues for the *C*-sulfonation of α,β-unsaturated carbonyl groups and are critical to the reaction mechanism.

### Expression analysis of SULT7A1

**Fig. 6.**
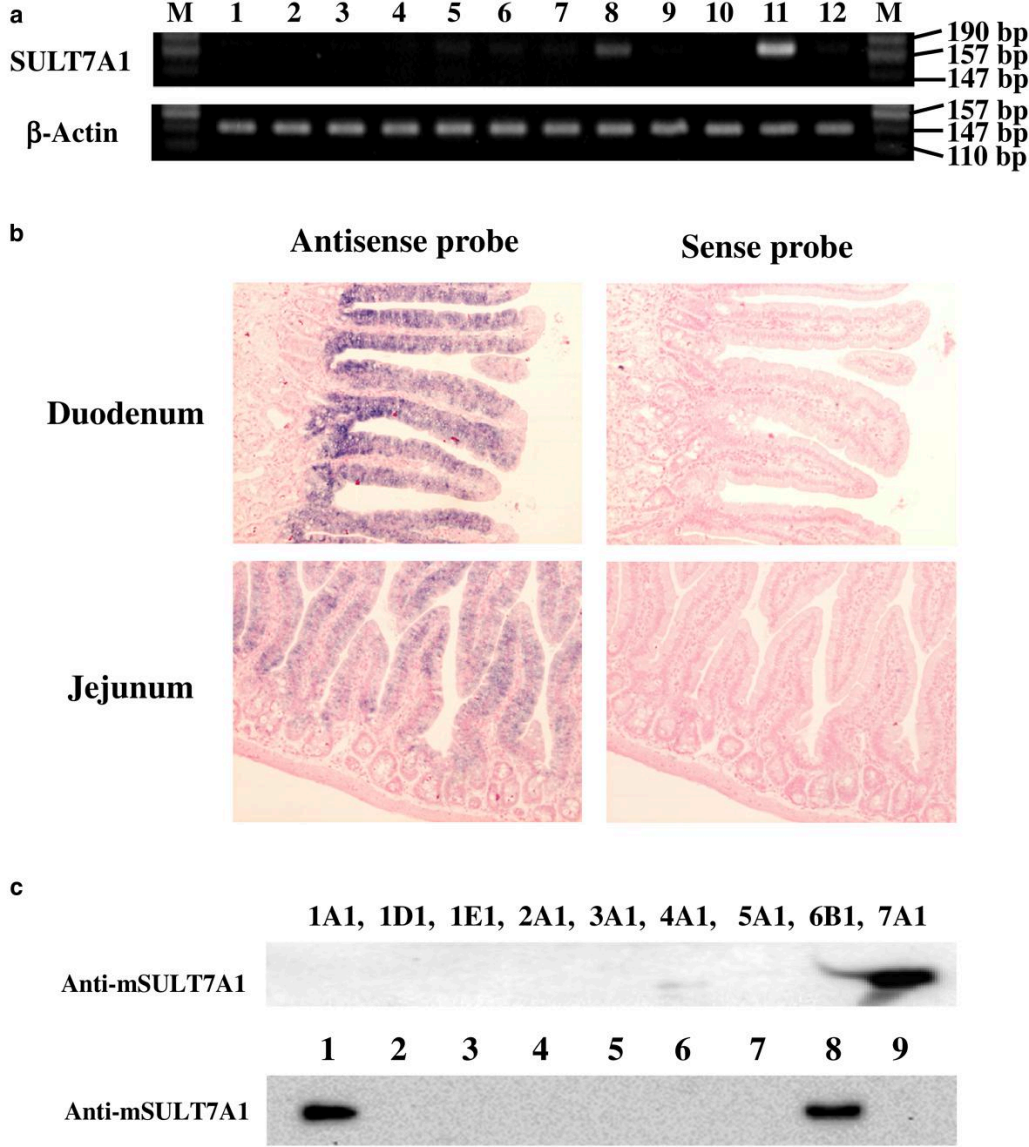
The expression of the prostaglandin-sulfonating SULT, SULT7A1. a) Tissue-specific mRNA expression of mouse SULT7A1 analyzed by RT-PCR. Lane 1: brain; lane 2: heart; lane 3: spleen; lane 4: kidney; lane 5: thymus; lane 6: lung; lane 7: skin; lane 8: stomach; lane 9: liver; lane 10: muscle; lane 11: small intestine; lane 12: testis. b) Cellular localization of SULT7A1 by *in situ* hybridization on mouse duodenal and jejunal sections. c) Protein expression of mouse SULT7A1. The upper panel shows the immunological cross-reactivity of anti-mSULT7A1 polyclonal antibody. Immunoblot analysis of 200 ng proteins of 9 different mouse recombinant SULTs (SULT1A1, SULT1D1, SULT1E1, SULT2A1, SULT3A1, SULT4A1, SULT5A1, SULT6B1, SULT7A1) was performed using rabbit polyclonal antibody against mSULT7A1. The lower panel shows the tissue-specific protein expression of mouse SULT7A1. Immunoblot analysis was performed with 10 μg cytosol from 8 different tissues. Lane 1: recombinant mSULT7A1; lane 2: brain; lane 3: heart; lane 4: kidney; lane 5: lung; lane 6: stomach; lane 7: liver; lane 8: small intestine; lane 9: testis.

The distribution of SULT in tissues/organs was analyzed at the mRNA and protein expression levels. RT-PCR analysis showed that message of SULT7A1 is specifically transcribed in the gastrointestinal tissues, particularly in stomach and small intestine among 12 tissues/organs tested (Fig. 6a). Further *in situ* hybridization analysis confirmed the specific and strong expression of SULT7A1 in the epithelial cells of duodenum and jejunum, consistent with the results of RT-PCR, while no expression was detected in other tissues including liver and kidney (Fig. 6b). In order to investigate the protein expression of SULT7A1, a polyclonal antibody was raised against mouse SULT7A1 enzyme, demonstrating high specificity with no cross activity toward other SULT enzymes. Western blotting experiment using the anti-SULT7A1 antibody supported the strong expression of SULT7A1 in the small intestine but not in the stomach (Fig 6c). It is therefore possible that SULT7A1 may be involved in the intestinal physiology through the sulfonation of α,β-unsaturated carbonyl compounds, particularly prostaglandins.

### Ligand assays of 15d-PGJ_2_ sulfonation products toward prostaglandin receptors

**Fig. 7.**
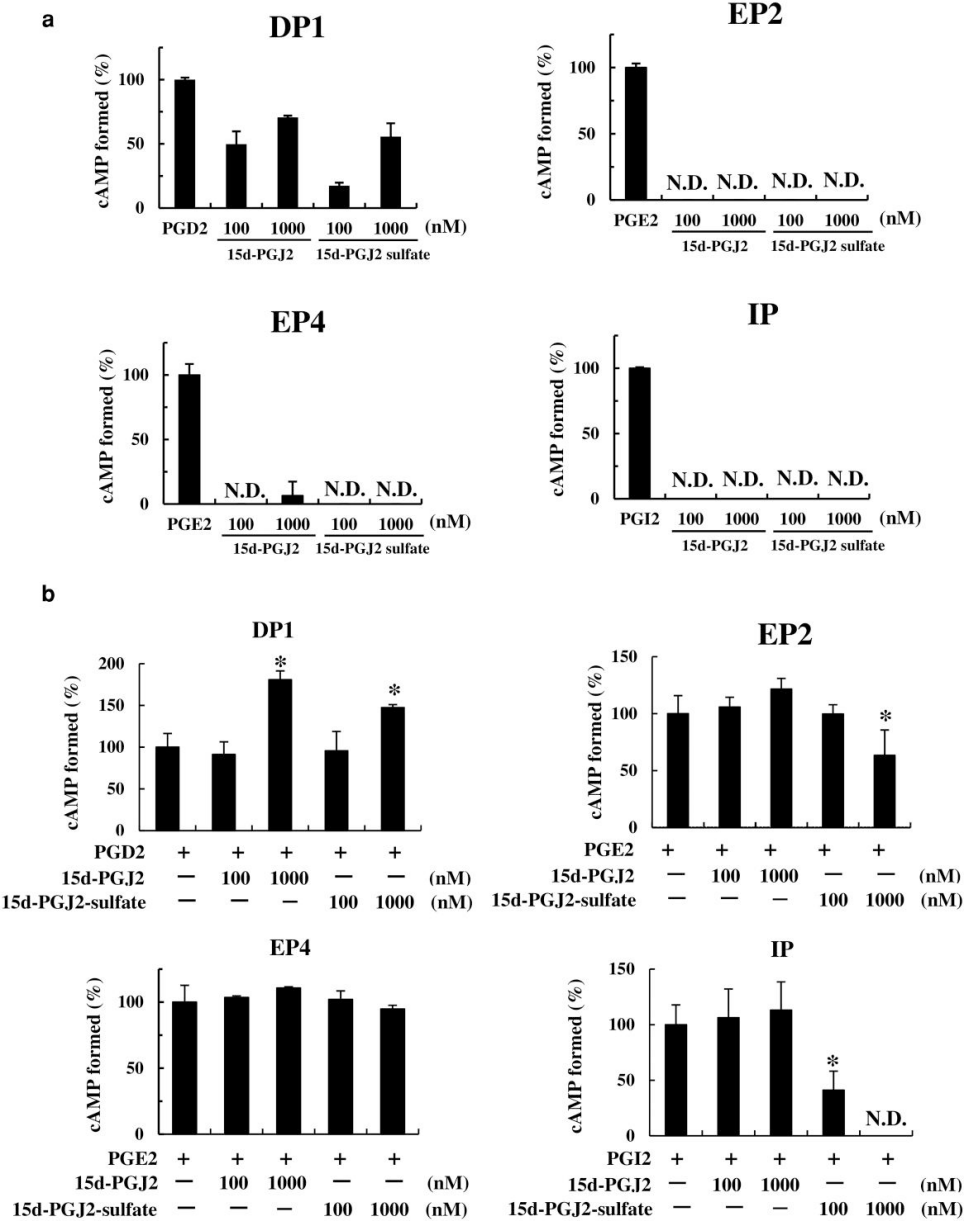
The effect of the sulfonation on the ligand activity of the 15d-PGJ_2_ toward prostanoid receptors. a) Agonist activity of sulfonated 15d-PGJ_2_ toward DP1, EP2, EP4, and IP. cAMP formed in cells in the presence of the corresponding ligand prostaglandins (10 nM), 15d-PGJ_2_ (100 or 1,000 nM), or sulfonated 15d-PGJ_2_ (100 or 1,000 nM) is expressed as relative values (%) against the amount of cAMP formed in the presence of the corresponding ligand prostaglandins. b) Antagonist activity of sulfonated 15d-PGJ_2_ toward DP1, EP2, EP4, and IP. cAMP formed in 293 T cells in the presence of the corresponding ligand prostaglandins (PGD_2_: 0.1 nM, PGE_2_: 0.05 nM, or PGI_2_: 10 nM) plus 15d-PGJ_2_ (100 or 1,000 nM), or sulfonated 15d-PGJ_2_ (100 or 1,000 nM) is expressed as relative values (%) against the amount of cAMP formed in the presence of the corresponding ligand prostaglandins. Statistical significance vs. the control sample (the corresponding ligand prostaglandins in the absence of 15d-PGJ_2_ and sulfonate 15d-PGJ_2_) is indicated by *P < 0.05 as analyzed by one-way ANOVA with Dunnett’s test. ND refers to the activity not detected.

To clarify the effect of sulfonation on 15d-PGJ_2_, sulfonated 15d-PGJ_2_ was examined for agonist and antagonist activities toward PGD_2_, PGE_2_, and PGI_2_ receptors (Fig. 7). Interestingly, sulfonated 15d-PGJ_2_ showed a weak agonist activity toward PGD_2_ receptor (DP) at relatively similar activity with 15d-PGJ_2_, implicating that sulfonation of 15d-PGJ_2_ does not alter the ligand activity toward DP1. Furthermore, 15d-PGJ_2_ and sulfonated 15d-PGJ_2_ showed no agonist activity toward PGE_2_ and PGI_2_ receptors (EP2, EP4, and IP). In contrast to the agonist activity, sulfonated 15d-PGJ_2_ exhibited the significant antagonist activity toward EP2 and IP, implying that sulfonation of 15d-PGJ_2_ may generate the physiological antagonists for EP2 and IP. These results implied that prostaglandin-modifying SULT7A1 may be involved in the regulation of the activity of cyclopentenone prostaglandin, 15d-PGJ_2_.

## Discussion

Phase II drug-metabolizing enzymes, including SULTs and glutathione-*S*-transferases (GSTs), are capable of modifying a variety of key endogenous compounds and altering the physiological functions (1–5, 32). GSTs have been demonstrated to modify α,β-unsaturated carbonyl-containing compounds including cyclopentenone prostaglandins (33, 34). However, the target groups for SULTs have long been recognized to be only hydroxy or amino group. The basic concept regarding the structure of substrate compounds for SULTs has thus lost sight of the potential substrates. In this study, the previously undescribed *C*-sulfonation of α,β-unsaturated carbonyl groups mediated by a newly identified mouse SULT7A1 is addressed, leading to the discovery that human SULT1C4 may be capable of catalyzing the *C*-sulfonation of α,β-unsaturated carbonyl groups.

The newly identified SULT7A1 belongs to a novel SULT family, SULT7, as none of the known SULT members shares >45% amino acid sequence identity with SULT7A1 (23). Alignment analysis also revealed that SULT7A1 lacks the dimerization motif KXXXTVXXXE, which is conserved in the p-loop of many SULT enzymes (35). Therefore, SULT7A1 may exist in a monomeric form, similar to mouse SULT1E1, SULT5A1, and SULT6B1 (36). Genome database searches have shown that orthologous genes of mouse *SULT7A1* are present in many mammals, birds, and amphibians in the NCBI genome database, as *SULT6B1-like* or *SULT6B2* (Fig. S10). However, in fish, the orthologous genes are found in only a few species, such as conger eel and lizard fish, but not in other teleost fish including zebrafish. The orthologous isoforms share >45% amino acid sequence identity with SULT7A1, as shown in Fig. S10. In primates, SULT7A1 genes are pseudogenes, except in the gray mouse lemur, and human *SULT7A1* pseudogene (gene ID: 107984497) is located in 12p12.1. The *SULT7A1* gene likely emerged during the evolution of fish but subsequently lost the enzymatic functions in the primates. Sequence alignment analysis with nine SULT7A1 orthologs revealed that all orthologs exhibit three catalytic residues, His^51^, His^94^, and Cys^234^, suggesting that the unique catalytic ability targeting α,β-unsaturated carbonyl groups is likely conserved in SULT7A1 (Fig. S10).

Each family and subfamily member has its own distinctive substrates. SULT1 family members are capable of catalyzing the sulfonation of the hydroxy group in simple phenolic compounds. *p-*Nitrophenol, dopamine, and 17β-estradiol are the representative substrates for SULT1A1, SULT1A3, and SULT1E1, respectively (37, 38). On the other hand, members of the SULT2 family are capable of sulfonating the hydroxy group of hydroxysteroids. Dehydroepiandrosterone and lithocholic acid are the representative substrates for SULT2A1, and cholesterol is the representative substrate for SULT2B1 (37, 38). In contrast, members of the SULT3 family display sulfonating activity toward the amino group of amines, such as naphthylamine and desipramine (39, 40). Since substrate specificity varies among different SULT families, it is anticipated that the newly identified SULT7A1 may utilize substrates with unique structural features that members of other SULT families do not recognize. Indeed, sulfotransferase assays demonstrated that SULT7A1 exhibited no sulfonating activity toward a wide range of previously identified substrate compounds containing the hydroxy or amino group. A different approach was, therefore, needed in order to identify the substrate compound(s) for SULT7A1. Then, a panel of compounds with diverse core structural components was examined to identify its substrates. SULT7A1, thus, exhibited specific sulfonating activity toward molecules containing α,β-unsaturated carbonyl groups, 2-cyclohexenone and 2-cyclopentenone. This result clearly indicated that SULT7A1 is a unique SULT enzyme which catalyzes a new type of the sulfonation reaction toward α,β-unsaturated carbonyl groups.

Cyclopentenone prostaglandins were identified as endogenous substrates for SULT7A1. It should be noted, however, that SULT7A1 is not capable of catalyzing the sulfonation of PGB_2_, implying that the secondary α- and β-carbons in the α,β-unsaturated carbonyl groups are crucial for the catalytic reaction. Among the four sulfonated cyclopentenone prostaglandins, SULT7A1 exhibited lower activity and affinity toward PGA_2_ compared with PGJ_2_ series. The alkyl chains of prostaglandins may influence the interaction between the enzyme's active site and the targeting group, α,β-unsaturated carbonyl group. SULT7A1 showed *K*_m_ values in the μM range for PGA_2_ and PGJ_2_ series, comparable with or smaller than those observed with GST enzymes (33). SULT7A1 may play an important role in the intestinal metabolism of cyclopentenone prostaglandins with GST enzymes. Indeed, metabolic labeling experiments with transiently SULT7A1-expressing cells have shown that SULT7A1 can be involved in the cellular prostaglandin metabolism. PGA_2_ is directly generated from PGE_2_ via chemical dehydration, while the conversion of PGD_2_ to PGJ_2_ series is more complicated (26, 27). PGD_2_ is converted into PGJ_2_ via chemical dehydration, which is followed by the additional chemical dehydration into15d-PGJ_2_ and the albumin-dependent metabolism into Δ^12^-PGJ_2_, respectively (26). In the metabolic labeling experiments, cells treated with PGD_2_ and PGJ_2_ generated two species of sulfonated metabolites. Upper spots may correspond to the sulfonated 15d-PGJ_2_ and lower spots may correspond to the sulfonated Δ^12^-PGJ_2_ or PGJ_2_ since those two species co-migrated with the sulfonated spots generated by *in vitro* enzymatic reaction (Fig. S11).

The *O*-sulfonation reaction, previously reported, has been proposed to proceed through an S_N_2-like in-line displacement mechanism (Fig. 1a), involving the transfer of a sulfonate group (–SO_3_H) from PAPS to a hydroxy group of the substrate (6, 7). The novel *C*-sulfonation reaction mediated by SULT7A1 may also proceed through an S_N_2-like reaction mechanism, involving the electrophilic β-carbon of α,β-unsaturated carbonyl group. MS analyses suggested that SULT7A1 catalyzes the transfer of a sulfonate group and one hydrogen atom to the α,β-unsaturated carbonyl group. NMR analyses confirmed that the sulfonate group is transferred to the α-carbon, and the β-carbon carries two hydrogen atoms. Crystal structure of SULT7A1 revealed a unique hydrogen bond formed between His^94^ and Cys^234^ in the active site. The flexible Cys^234^-containing loop is likely the key region for the substrate acceptance and catalysis. Previous studies have shown that this loop and a segment are important regions forming the substrate entry gate for other SULT enzymes, referred to as loop-1 and segment-1, respectively (41, 42). It should be noted that SULT7A1 lacks the conserved segment-1 region, as shown in Fig. 5 and Fig. S2 (dashed line area). Deletion of Cys^234^ resulted in the formation of an alternative substrate entry gate by the flexible loop-1 (Fig. S9a). These observations imply that the flexibility of loop-1 may be crucial for the docking of substrates into the active site, and the docking mechanism of a substrate differs from that of other SULTs. Substrates may dock into the active site through the substrate route formed by the alternative segment-1 and α-helix from Thr^50^ to Ile^60^, which includes His^51^ (Figs. 5b and S2). The loop-1 may also serve as a guide to introduce substrates to the substrate route. Molecular dynamics simulation analysis was performed to investigate the possible conformation of the loop-1 of SULT7A1 (Fig. S9b). The simulation analysis suggested that the conformation of loop-1 may exist in both open and closed state due to its high flexibility. While no substrate entry gate was observed in the entire structure of all simulated models, a larger entry gate and a tunnel connecting to the active site appeared in the simulated open models (Fig. S9c). Therefore, the flexible loop may create a pocket for substrate docking into the active site. Docking simulation analysis also suggested that 15d-PGJ_2_, a representative substrate, can dock from the gate formed by the open loop into the active site (Fig. S9d). Additionally, His^51^ may play a catalytic role in addition to His^94^ and Cys^234^. Docking a substrate in the pocket may cause the release of Cys^234^ from His^94^ in the flexible loop. Interestingly, SULT7A1 failed to catalyze the sulfonation of other α,β-unsaturated carbonyl compounds, ketosteroids and naphthoquinones. These results implied that SULT7A1 selectively recognizes the alkyl chains of prostaglandins, and the alkyl chains may play an important role in the formation of flexible loop. It is noted that α,β-unsaturated carbonyl-containing compounds are known to undergo nucleophilic attack at the β-carbon by a variety of nucleophiles such as enolate anions, amines, or thiols (43, 44). A hypothetical catalytic mechanism thus involves the docking of a substrate compound into the active site, leading to the release of Cys^234^ from His^94^ (Fig. S12). Some residues, including His^51^, may catalyze the electron transfer to the electrophilic β-carbon of the substrate compound. The donated electron subsequently assists in the formation of an enol intermediate, and the α-carbon then makes a nucleophilic attack at the sulfur atom of PAPS. Although substrate soaking experiments were also conducted to understand the mechanism of the initiation of the enol intermediate formation, no crystal structure was obtained in this study. Further experiments are required to explore this unique *C*-sulfonation reaction mechanism.

It is an important point to determine whether other SULTs are capable of catalyzing this novel reaction. Thus, 13 human SULT enzymes were examined in the enzymatic assays with 2-cyclopentenone, 2-cyclohexenone, and 15d-PGJ_2_. Among 13 SULTs, SULT1C4 exhibited strong sulfonating activity toward 2-cyclohexenone, but not 2-cyclopentenone and 15d-PGJ_2_ (Table S2). This result implies that, in human, sulfonation of α,β-unsaturated carbonyl groups may occur for six-membered ring-containing compounds, but not for five-membered ring-containing compounds. Additionally, sulfatase assays toward the sulfonated products revealed that sulfonated 2-cyclohexenone, generated by hSULT1C4 and mSULT7A1, is tolerant to the sulfatase reaction, in contrast to the sulfonated phenol (Fig. S13). This suggests that human SULT1C4 may catalyze the transfer of the sulfonate group to α-carbon of α,β-unsaturated carbonyl groups. Even though most primates lack the functional SULT7A1 orthologs, SULT1C4 may compensate the *C*-sulfonation of α,β-unsaturated carbonyl groups. While the catalytic residue His^94^ is conserved among all SULTs, neither His^51^ nor Cys^234^ is observed in other SULTs, including hSULT1C4, with the exception of Cys^230^ in SULT4A1 (Fig. S2). A previous paper reported that structure of SULT4A1 lacks in the loop, as shown in Fig. S2. Additionally, Cys^230^ is located in α-helix (45), making it unlikely for SULT4A1 to form a hydrogen bound between His and Cys. While the reaction mechanism for the sulfonation of α,β-unsaturated carbonyl groups by hSULT1C4 is still unresolved, a key point of the novel sulfonation may be the generation of a nucleophilic atom capable of attacking the sulfur atom of PAPS. Interestingly, in the *O*-sulfonation of ketosteroids, it is proposed that SULT2A1 deprotonates the C-6 proton of ketosteroids though the catalytic residue His, forms an enolate intermediate, and generates a nucleophilic oxyanion which attacks the sulfur atom of PAPS (15). The difference between *O*-sulfonation and *C*-sulfonation may be due to the stability of oxyanion, which is usually unstable. Further studies are warranted in order to clarify the mechanism of these new reactions.

Another issue is related to the physiological relevance of the *C*-sulfonation of α,β-unsaturated compounds, including cyclopentenone prostaglandins, mediated by SULT7A1. Expression analyses of mRNA and protein showed that SULT7A1 is specifically expressed in the epithelial cells of the small intestine. Prostaglandins are known to be involved in the intestinal homeostasis including the barrier function, immune system, and inflammatory (46–48). On the other hand, cyclopentenone prostaglandins, chemical metabolites of prostaglandins, exhibit the anti-inflammatory effects through the peroxisome proliferator-activated receptor γ (27). Many studies have suggested that both the inflammatory PGE_2_ and anti-inflammatory 15d-PGJ_2_ are important in the physiological homeostasis of prostaglandins, and the disruption of this balance may lead to the cancer diseases (49, 50). Therefore, antagonist effect of sulfonated 15d-PGJ_2_ on EP2 may enhance the anti-inflammatory effects of 15d-PGJ_2_ by inhibiting the inflammatory effects of PGE_2_. IP is the specific receptor for PGI_2_, which plays an important role in the vascular homeostasis such as platelet inhibition and vasodilation (51, 52). Failure in vascular homeostasis may cause the intestinal ischemia, leading to inflammatory bowel disease (IBD) (53). IP has also been suggested to regulate the acute inflammation and immune system through the receptor response (51, 54). The antagonist effect of sulfonated 15d-PGJ_2_ on IP may regulate the intestinal vascular homeostasis and inflammation. It is therefore possible that SULT7A1 may be involved in the intestinal physiology through the sulfonation of α,β-unsaturated carbonyl compounds, including prostaglandins and other potential physiologically active substrates.

To summarize, the present study unequivocally demonstrated a new type of sulfonation reaction (*C*-sulfonation), catalyzed by the newly discovered prostaglandin-modifying enzyme, SULT7A1. SULT7A1 may play a crucial role in regulating prostaglandin function through the modification of cyclopentenone prostaglandins. Further studies are warranted in order to explore the physiological relevance of the prostaglandin modification through sulfonation. The novel *C*-sulfonation reaction underscores the importance of broadening the conception regarding the spectrum of potential substrate compounds for SULTs. It is possible that the sulfonation targeting the α,β-unsaturated carbonyl groups is mediated not only by SULT7A1 but also by other SULT enzymes. Indeed, human SULT1C4 exhibits the strong sulfonating activity toward an α,β-unsaturated carbonyl compound, 2-cyclohexenone. Therefore, human SULT1C4 may be involved in the metabolism of other α,β-unsaturated carbonyl compounds, such as quinones. Additionally, our recent work showed that SULT2A1 is capable of catalyzing the *O*-sulfonation of ketosteroids in a manner distinct from that catalyzed by SULT7A1 (15). The proposed reaction mechanisms found for α,β-unsaturated carbonyl groups are different between *O*-sulfonation and *C*-ulfonation, implying that sulfonation of α,β-unsaturated carbonyl groups may be just one of the ways to generate a nucleophile attack at the sulfur atom of PAPS. Additional yet-unidentified routes leading to similar nucleophile attacks may exist for the sulfonation of the wide variety of endogenous metabolites and xenobiotics encountered in the body. SULT4A1 and SULT6B1, referred to as orphan SULTs (55, 56), have been reported to exhibit very weak sulfonating activities (57, 58). Therefore, attention may need to be directed toward other types of substrates that do not contain hydroxy or amino groups in their structures. Undoubtedly, more studies are warranted to comprehensively elucidate the sulfonation pathway, encompassing not only the chemistry but also the physiological implications of SULT-mediated sulfonation.

## Materials and methods

### Molecular cloning and preparation of expression constructs of a novel mouse sulfotransferase SULT7A1

A novel mouse *SULT* gene (gene ID: 330440), designated *SULT7A1*, was discovered in the mouse genome database (NCBI). The identified mouse *SULT7A1* nucleotide sequence was amplified by RT-PCR. With the mouse small intestine total RNA (OriGene Technologies, Rockville, MD, USA) as the template and oligo(dT) as the primer, the first-strand cDNA was synthesized using a First-Strand cDNA Synthesis Kit (TOYOBO, Osaka, Japan). To clone the ORF of mouse SULT7A1, a PCR, using sense and antisense oligonucleotide primers (Table S3), in a 20-μL reaction mixture was carried out under the action of KOD-plus- (TOYOBO), with the first-strand cDNA prepared as template. Amplification conditions were 2 min at 94 °C, followed by 30 cycles of 30 s at 94 °C, 30 s at 60 °C, 1.5 min at 68 °C. The amplified DNA fragments were subcloned into the *Bam*HI/*Eco*RI site of pBluescript II SK(+) and transformed into *Escherichia coli* XL1-Blue MRF′ (Stratagene, Tokyo, Japan). To verify their authenticity, the cDNA inserts were subjected to nucleotide sequencing. To obtain the full-length cDNA sequence of mouse SULT7A1, 5′-rapid amplification of cDNA ends (RACE) and 3′-RACE were performed under the action of LA *Taq* (Takara Bio, Ohtsu, Japan). The 5′-RACE was carried out using a 5′-Full RACE Core Set (TAKARA) according to the manufacturer's protocol with a gene-specific primer (Table S3) for the RT reaction. First PCR was performed using a gene-specific sense primer and a gene-specific antisense primer (Table S3) with circular single-stranded cDNA as template. Amplification conditions were 10 cycles of 30 s at 95 °C, 30 s at 60 to 50 °C (a decrease of 1 °C per cycle), 1.5 min at 72 °C, followed by 25 cycles of 30 s at 95 °C, 30 s at 50 °C, 1.5 min at 72 °C. The nested PCR was carried out using a gene-specific sense and antisense primer (Table S3). Amplification conditions were 3 min at 95 °C, followed by 25 cycles of 30 s at 95 °C, 30 s at 52 °C, 1.5 min at 72 °C. For the 3′-RACE, RT-PCR technique was employed with an anchored oligo(dT) antisense primer (Table S3) for RT reaction. First PCR was then performed using a gene-specific sense primer and an anchored primer (Table S3). Amplification conditions were 10 cycles of 30 s at 94 °C, 1 min at 55 to 45 °C (a decrease of 1 °C per cycle), 2.5 min at 72 °C, followed by 25 cycles of 30 s at 94 °C, 30 s at 55 °C, 2.5 min at 72 °C. The semi-nested PCR was carried out using a gene-specific sense primer (Table S3), and the anchored primer used for first PCR. Amplification conditions were 30 s at 94 °C, followed by 25 cycles of 30 s at 94 °C, 30 s at 55 °C, 2.5 min at 72 °C. The amplified DNA fragments were subcloned into pBluescript II SK(+) and verified by sequencing. To generate the expression construct containing mouse SULT7A1 cDNA, the ORF cloned in pBluescript was digested by *Bam*HI/*Eco*RI (NEB Japan, Tokyo, Japan) and subcloned into the *Bam*HI/*Eco*RI site of pGEX-4T-1 prokaryotic expression vector (GE Healthcare Japan, Tokyo, Japan) or pEF6/V5-His mammalian expression vector (Invitrogen, Tokyo, Japan).

### Site-directed mutagenesis

Site-directed mutagenesis was performed using mutagenic primers, encoding specific amino acid mutations in mouse SULT7A1, to obtain mutants: H51A, H94A, and C234A (Table S3). Wild-type SULT7A1 cDNA packaged in pGEX-4T-1 was used as the template in PCRs. Amplification conditions used were 2 min at 94 °C, followed by 12 cycles of 20 s at 94 °C, 1 min at 55 °C, and 6 min at 68 °C with KOD-plus-Neo (TOYOBO). Mutated SULT7A1 packaged in pGEX-4T-1 was verified by nucleotide sequencing, treated with *Dpn*I for 12 h at 37 °C, and used to transform *E. coli* XL1-Blue MRF′ cells.

### Bacterial expression and purification of the recombinant proteins

pGEX-4T-1 harboring the mouse SULT7A1 cDNA was transformed into competent *E. coli* BL21 cells. Transformed BL21 cells were grown to OD_600 nm_ = ∼0.5 in 200 mL Luria Bertani (LB) medium supplemented with 100 μg/mL ampicillin, and induced with 0.1 mM isopropyl β-D-thiogalactopyranoside (IPTG) for 12 h at 24 °C. Recombinant mouse SULT7A1 was purified from the homogenate of IPTG-induced BL21 cells based on a previously developed procedure (59). Briefly, the collected cells, in ice-cold lysis buffer (50 mM Tris-HCl at pH 8.0, 150 mM NaCl, 1 mM EDTA), were homogenized with an Ohtake French Press. The supernatant of homogenate was prepared by the centrifugation (20,400 *× g* for 20 min) and subjected to the affinity chromatography using glutathione–sepharose resin (GE Healthcare Japan). The purified recombinant protein was then released from the resin by thrombin digestion for 3 h at 4 °C. Protein concentration of purified mouse SULT7A1 was determined using Lowry's method, with bovine serum albumin as the standard. The purity of recombinant SULT7A1 was judged to be >95% in purity based on SDS–PAGE on a 12% polyacrylamide gel using method of Laemmli. For human SULTs, 13 recombinant enzymes (1A1, 1A2, 1A3, 1B1, 1C2, 1C3a, 1C3d, 1C4, 1E1, 2A1, 2B1b, 4A1, 6B1) were prepared by the same procedure (59).

### Enzymatic assay

Sulfotransferase activity was assayed using [^35^S]-PAPS as the sulfonate donor, which was synthesized from ATP and [^35^S]-sulfate by using recombinant human bifunctional ATP sulfrylase/adenosine 5′-phosphosulfate kinase, as described previously (60). The standard assay mixture, with a final volume of 25 μL, contained 50 mM sodium phosphate buffer at pH 7.5, 0.4 μM [^35^S]-PAPS (45 Ci/mmol), 0.2 mM DTT, and 1–1,000 μM of tested substrate. Controls with DMSO, in place of the substrate, were also prepared in parallel. The reaction was started by the addition of 0.5 μg purified SULT enzymes, allowed to proceed for 10 min at 30 °C (SULT7A1) or 37 °C (human SULTs), and terminated by heating at 100 °C for 3 min. The precipitates formed were cleared by centrifugation, and the supernatant was subjected to the analysis of [^35^S]-sulfonated products using a cellulose thin-layer chromatography (TLC) procedure (24), with *n*-butanol/isopropanol/formic acid/water (3:1:1:1; by volume) as the solvent system. Afterwards, autoradiograph was taken from the TLC plate (Merck, Darmstadt, Germany), and the radioactive spots corresponding to [^35^S]-sulfonated products of compounds tested were quantitatively determined using a fluorescent image analyzer Typhoon FLA9500 (GE Healthcare Japan). The results obtained were calculated and expressed in nanomoles of sulfonated product formed/min/mg purified enzyme. For sulfatase assay, 10 μL sulfotransferase reaction mixtures containing phenol, 2-cyclopentenone, or 2-cyclohexenone as substrates were subjected to the 20 min reaction, followed by the addition of 10 mU *Aerobacter aerogenes* sulfatase and incubation for 3 h at 37 °C. The reaction supernatants, terminated by heating at 100 °C for 3 min, were analyzed by the procedure described above.

### MS and NMR analyses of sulfonated products

To prepare sulfonated 2-cyclopentenone (Sigma-Aldrich, Tokyo, Japan) and 15d-PGJ_2_ (Cayman Chemical, Ann Arbor, MI, USA) for MS and NMR analyses, the above-mentioned sulfotransferase reaction was performed at larger scale with nonradioactive PAPS and an extended reaction time (3 h). For the reaction using 2-cyclopentenone, the reaction mixture was applied on to an Oasis WAX cartridge (Waters, Milford, MA, USA), and the sulfonated product was eluted using 1% NH_4_OH/99% methanol. The eluate was dried, reconstituted in methanol, and analyzed using a Q-Exactive hybrid quadrupole-orbitrap mass spectrometer (Thermo Fisher Scientific, Waltham, MA, USA) with a heated electrospray ionization source generated by direct infusion. Typical mass spectrometric analysis conditions were: polarity, negative ionization mode; spray voltage 2.0 kV; sheath gas flow rate, 10; heated capillary temperature, 270 °C. For the reaction using 15d-PGJ_2_, the reaction mixture was fractionated using a Sep-Pak C18 (Waters). The sulfonated product was eluted using water/methanol (3:2, by volume) and further separated by HPLC using a Shimadzu Prominence HPLC system fitted with a photodiode array detector. A 5-μm Capcell PAK C18 MGII column (4.6 × 250 mm; SHISEIDO, Tokyo, Japan) maintained at 40 °C was used, and a gradient elution using 0.05% TFA buffer (in H_2_O) and methanol at a flow rate of 1 mL/min for 50 min was applied. The methanol concentrations used were: 0% (0–5 min), 0–60% (5–10 min), 60–100% (10–40 min), 100% (40–45 min), 100–0% (47–52 min), and 0% (50–60 min). Sample isolated at 20–22 min retention time was dried, reconstituted in methanol, and analyzed by mass spectrometer as mentioned above. For NMR analysis, sulfonated 15d-PGJ_2_ were dissolved in D_2_O and DMSO-d6 (Sigma-Aldrich), respectively, and analyzed using a Bruker Advance 400 instrument (400 MHz, 9.4T; Bruker, Tokyo, Japan). Data obtained are summarized in Table S1.

### Metabolic labeling of mouse SULT7A1 overexpressing BHK-21 hamster kidney cells

BHK-21 cells (obtained from the Cell Resource Center for Biomedical Research Institute of Development, Aging and Cancer, Tohoku University) were routinely maintained in α-Minimum Essential Medium (MEMα; FUJIFILM Wako Pure Chemical, Osaka, Japan) supplemented with 10% fetal bovine serum, 100 U/mL penicillin, and 100 mg/mL streptomycin sulfate at 37 °C and 5% CO_2_. BHK-21 cells, grown to 80% confluence in individual wells of a 24-well culture plate, were transfected with pEF6 or pEF6-SULT7A1 plasmid using Lipofectamine 2000 (Invitrogen) based on standard procedures. Transfected cells were maintained in the above-mentioned culture medium. After a 36-h incubation, the cells were preincubated in sulfate-free MEM (S-MEM JOKLIK; Irvine Scientific, Santa Ana, CA, USA) for 6 h, and labeled with 200 μL aliquots of the same medium containing [^35^S]-sulfate (50 μCi/mL), and 25 μM of tested prostaglandin. At the end of a 15-h labeling, the media were collected and subjected to the analysis of [^35^S]-sulfonated products using TLC with ethyl acetate/methanol/water (8:2:1; by volume) as the solvent system. Upon completion of TLC, an autoradiograph was taken from TLC plate to reveal radioactive spots corresponding to [^35^S]-sulfonated products of tested compounds added to the labeling media.

### Crystallization and data collection

SULT7A1 recombinant protein was purified by the above-mentioned methods. Purified SULT7A1 was adjusted to 8 mg/mL in 50 mM Tris-HCl, pH 7.9, 150 mM NaCl, 10 mM dithiothreitol, and 5 mM PAP. Crystals of SULT7A1 were prepared by the hanging-drop vapor diffusion method at 20°. Hanging drops contained 1 μL of protein mixed with 1 μL of reservoir solution (16% polyethylene glycol (PEG) 10,000, 10 mM dithiothreitol and 100 mM Bis–Tris, pH 5.5). Crystals were grown for 3 days. Prior to data collection, crystals were transferred to a cryoprotectant solution consisting of 150 mM NaCl, 16% PEG 10,000, 5 mM PAP, 10 mM dithiothreitol, 100 mM Bis–Tris, pH 5.5, and 25% glycerol and flash cooled to −180 °C. X-ray diffraction data were collected at the beamline BL38B1 of Spring-8 (Hyogo, Japan). Diffraction data were processed using the program package HKL2000 (HKL Research Inc., Charlottesville, VA, USA) (61). The structure was determined by molecular replacement, using the program Molrep (62). The crystal structures of mouse SULT1D1 were used as the search models. Model building was carried out with the program Coot (63). The program Refmac5 (64) was used for refinement. Refinement statistics are summarized in Table S4. Structure quality was assessed using PROCHECK (65). The atomic coordinates of SULT7A1 are deposited in Protein Data Bank under accession number 5X2B.

### mRNA expression analysis by RT-PCR and in situ hybridization

For use as templates in RT-PCR, first-strand cDNAs were reverse-transcribed from total RNA isolated from 12 mouse organs, purchased from OriGene Technologies, using a First-Strand cDNA Synthesis Kit (TOYOBO). Total RNAs used as template included mixture of three male and female BALB/C mice 6–8 weeks old, per organs/tissues, except for testis. Using a gene-specific sense and antisense oligonucleotide primers (Table S3), PCRs in 20 μL reaction mixtures were carried out under the action of *Taq* DNA polymerase, with each of the 12 (brain, heart, spleen, kidney, thymus, lung, skin, stomach, liver, muscle, small intestine, and testis) first-strand cDNAs prepared as template. Reaction conditions were 3 min at 95 °C for initial denaturation, followed by 25 cycles of 30 s at 95 °C, 30 s at 50 °C, and 60 s at 72 °C. The final reaction mixture was applied onto a 2.5% agarose gel, separated by electrophoresis, and visualized by ethidium bromide staining. For in situ hybridization, formalin-fixed and paraffin-embedded sections (4 μm thick), harvested from male C57BL/6 mice 10-week age, were fixed in 4% paraformaldehyde in phosphate-buffered saline (PBS). The sections were air-dried and rinsed with nuclease-free water and used for the subsequent in situ hybridization experiments. To generate digoxigenin-labeled RNA probes, a 873-bp cDNA fragment corresponding to ORF sequence of mouse SULT7A1 was used as a template for in vitro transcription, according to the manufacturer's instructions (Roche Diagnostic KK, Tokyo, Japan). Same amount of antisense or sense (as a negative control) probe was used for hybridization. *In situ* hybridization reaction was performed using a fully automated in situ hybridization apparatus (Ventana HX System Discovery and RiboMap System, Roche Diagnostic KK), according to manufacturer's instruction. Briefly, following pretreatment (fixation, acid treatment, and conditioning without protease treatment), the sections were subjected to hybridization using 500 ng per slide of digoxigenin-labeled probe at 65 °C for 6 h. After hybridization, signals were detected with biotin-labeled anti-DIG antibody. The reaction was visualized using a BlueMap Kit, and counterstaining was performed using nuclear fast red. The use of mice in this experiment was approved by University of Miyazaki, Institutional Animal Care and Use Committee.

### Protein expression analysis by immunoblotting

Mouse organs (brain, heart, kidney, lung, stomach, liver, small intestine, testis), harvested from two male BALB/C mice 10-week age, rinsed thoroughly with ice-cold PBS, and suspended in PBS buffer supplied with 1 mM PMSF and a protease inhibitor cocktail (Roche Diagnostic KK), were homogenized using a ULTRA-TURRAX T8 (IKA, Staufen, Germany). Homogenized preparations were centrifuged at 100,000 × *g* for 1 h at 4 °C, and the resulting supernatants were used in the immunoblotting. Supernatant samples (10 μg) were mixed with a 2× SDS sample buffer, followed by heating. Afterwards, the samples were subjected to SDS–PAGE and electroblotted onto an Immobilon-P membranes (Millipore, Tokyo, Japan). The blotted membrane was blocked with 5% nonfat milk in PBS with 0.1% Tween-20 for 1 h, probed with rabbit anti-mSULT7A1 polyclonal antibody at a dilution of 1:200 overnight at 4 °C or a monoclonal mouse anti-β-actin antibody (Sigma-Aldrich) at a 1:5,000 for 1 h, washed with PBS containing 0.1% Tween-20, and incubated with antirabbit IgG horseradish-peroxidase-linked antibody (Cell Signaling Technology, Danvers, MA, USA) at 1:1,000 for 1 h. Polyclonal antibody against mouse SULT7A1 was raised in rabbit, and the antibody therein was affinity-purified using purified recombinant mSULT7A1 covalently bonded to Affi-Gel 10 Gel (Bio-Rad, Hercules, CA, USA), according to manufacturer's instructions. Purified antibody was stored in 50% glycerol solution at 0.2 mg/mL. Immunoreactive bands were visualized using the ECL Plus detection system (GE Healthcare Japan), according to the manufacturer's instructions, and detected using ChemiDoc XRS (Bio-Rad). The use of mice in this experiment was approved by University of Miyazaki, Institutional Animal Care and Use Committee.

### cAMP assay using 293T human embryonic kidney cells transiently expressing prostanoid receptors

cDNA sequences of PGD_2_ receptor (DP1), PGE_2_ receptor-2/-4 (EP2 and EP4), and PGI_2_ receptor (IP) were amplified using first-strand cDNA prepared from intestine total RNA as a template by RT-PCR as described in the section of Molecular Cloning of SULT7A1. Gene-specific oligonucleotide primer sets were used to amplify cDNA sequences of DP1, EP2, EP4, and IP (Table S3). The generated cDNAs, upon verification by nucleotide sequencing, were subcloned into the *Eco*RI/*Xba*I site of the pcDNA4 mammalian expression vector (Invitrogen). The plasmid constructs prepared were individually transfected into 293T human embryonic kidney cells (ATCC, Manassas, VA, USA), routinely maintained in DMEM (FUJIFILM Wako Pure Chemical) supplemented with 10% fetal bovine serum, 100 U/mL penicillin, and 100 mg/mL streptomycin sulfate at 37 °C and 5% CO_2_, in individual wells of a 24-well culture plate with Lipofectamine LTX (Invitrogen). After a 24-h incubation, culture media were replaced with PBS and cells were pretreated with 500 μM isobutyl-1-methylxanthine and 100 μM 4-(3-butoxy-4-methoxy-benzyl) imidazolidone for 15 min. Prostaglandins (PGD_2_, PGE_2_, PGI_2_, 15d-PGJ_2_, and sulfonated 15d-PGJ_2_) were individually added to cells in different wells, and incubated for 15 min. Afterwards, cyclic AMP (cAMP) level was analyzed using a cAMP-Glo Assay kit (Promega Japan, Tokyo, Japan), according to the manufacturer's instructions, and measured using a Luminescencer-PSN (Atto, Tokyo, Japan).

## Supplementary Material

pgae097_Supplementary_Data

## Data Availability

All data are included in the article and Supplementary material.
