# Supplementary material for: A new type of sulfation reaction: C-sulfonation for α,β-unsaturated carbonyl groups by a novel sulfotransferase SULT7A1
Source: PNAS Nexus. 2024 Mar 4;3(3):pgae097. doi: 10.1093/pnasnexus/pgae097 (PMC10939482; doi:10.1093/pnasnexus/pgae097)
Supplement: pgae097_Supplementary_Data [file pgae097_supplementary_data.pdf]

A new type of sulfation reaction: *C*-sulfonation for α,β-unsaturated carbonyl groups by a novel sulfotransferase

Katsuhisa Kurogi[1]*, Yoichi Sakakibara[1], Takuyu Hashiguchi[1], Yoshimitsu Kakuta[2], Miho Kanekiyo[2], Takamasa Teramoto[2], Tsuyoshi Fukushima[3], Takeshi Bamba[4], Jin Matsumoto[5], Eiichiro Fukusaki[6], Hiroaki Kataoka[3], and Masahito Suiko[1]

Contents

Page S-1: Figure S1
Page S-2: Figure S2
Page S-3: Figure S3
Page S-4: Figure S4
Page S-5: Figure S5
Page S-6: Figure S6
Page S-7: Figure S7
Page S-8: Figure S8
Page S-9: Figure S9
Page S-10: Figure S10
Page S-11: Figure S11
Page S-12: Figure S12
Page S-13: Figure S13
Page S-14: Table S1
Page S-15: Table S2
Page S-16: Table S3
Page S-17: Table S4

Figure S1

```
     AGAGATACCAAGATTCCTCTGCCACTCTGCTGAGGTTGTGACCAAGGGCCAGGTTTGTCT   60
     GTGGCTTGTGGAGGGACTGTGCTGACTATACAGCCTCTAATAGGGTCAGTGGAAGATGTC  120
  1                                                         M  S
     ATCCCAGAGTCAGCCAAGTCTCCTCCATAAGTACATGGGCATCTTCTTCTCTACCATGTC  180
  3   S  Q  S  Q  P  S  L  L  H  K  Y  M  G  I  F  F  S  T  M  S
     TTCAGAAGAACTCCTTGGTTCCTTGGACTCTTTTGATGCTAGAGAAGATGACATATTTCT  240
 23   S  E  E  L  L  G  S  L  D  S  F  D  A  R  E  D  D  I  F  L
     GGTTTCCTACCCAAAATCTGGCACTCATTGGCTGGCAGAAGTCATTGAGCGCATTCCCGA  300
 43   V  S  Y  P  K  S  G  T  H  W  L  A  E  V  I  E  R  I  P  D
     CGCTGGCATCACACTCACTTCTCCTATTGAATTGGGAGACATTTCTAAATTTGAAGAGCT  360
 63   A  G  I  T  L  T  S  P  I  E  L  G  D  I  S  K  F  E  E  L
     GAAAAGGATACCTAAGAGAAGAGCTATCCCGACACATCTGAACTATGAAATGCTTCCTGT  420
 83   K  R  I  P  K  R  R  A  I  P  T  H  L  N  Y  E  M  L  P  V
     GACTGTGAAACAGAAGCAGTGCAAGATTATCTACATCGTCAGAAATCCGAAGGATACAGC  480
103   T  V  K  Q  K  Q  C  K  I  I  Y  I  V  R  N  P  K  D  T  A
     CGTCTCCATGTTCCACTACTACAGGGACAACCCCAATCTCCCTTCCACGGAAACATGGGC  540
123   V  S  M  F  H  Y  Y  R  D  N  P  N  L  P  S  T  E  T  W  A
     TGCGTTTTTAGAGCTGTTCCTCAAAGGAGATGTTGTGTATGGTTCTTGGTTTGATCACGT  600
143   A  F  L  E  L  F  L  K  G  D  V  V  Y  G  S  W  F  D  H  V
     TTTGAGTTGGGAAGAACACAAAAACGATAAAAATGTCCTATTCATCTTCTATGAAGAAAT  660
163   L  S  W  E  E  H  K  N  D  K  N  V  L  F  I  F  Y  E  E  M
     GAAAAAAGATTTTGTTAAGAGCCTAAAGAAAATAACTGCTTTCCTCGGCATCGACGTGAA  720
183   K  K  D  F  V  K  S  L  K  K  I  T  A  F  L  G  I  D  V  N
     TGACAGCGAGATGGCTAAGATCGCTCGGAGTACGTCATTCAGTGAAATGAAAAGTAATGC  780
203   D  S  E  M  A  K  I  A  R  S  T  S  F  S  E  M  K  S  N  A
     AGCCAAAGAAAATTGTGATCCCAATCACGTCATCTGTGCCCTCACGTCCGACAGGAACCT  840
223   A  K  E  N  C  D  P  N  H  V  I  C  A  L  T  S  D  R  N  L
     GGTGTTCAGAAAAGGAGTGGTGGGTGATTGGATAAACTACTTCACTCCGAAGCAGAACAG  900
243   V  F  R  K  G  V  V  G  D  W  I  N  Y  F  T  P  K  Q  N  R
     AGGCTTTGATGAACTATTCACGGAGAAGATGAGAAACAGTGACGTGGGCAGATGCTTGAA  960
263   G  F  D  E  L  F  T  E  K  M  R  N  S  D  V  G  R  C  L  K
     GGAGTATGCGCATTCGCAGAATGCGTGATGGAGTACGCGCAATCGCAGATGCATGAAGGA 1020
283   E  Y  A  H  S  Q  N  A  *
     GTGCGCGCAATCAGATGGGATGCGAAGTGGATTTCGTTCTCTGCCGCGTGGCGCCTGGGA 1080
     AACATGTAAATATGCTTTATTGATGTGGAAAGCAGATAGTTCTTTCCGTGAAAAAGACGT 1140
     ACTTCAAAAATGTGTCAAAAGCGGCACTCACGAGACTCTACTATCATATTTGGTGACAGC 1200
     AGAATTATTAATAAAGGGGGGCATAAAATTCTCCAGTCGTTGAACTCTGTTCAGTTCATT 1260
     TTCAGCGAGTTCTGCTGGTTCCTATGCAGAAAGAGCGGTTATTGCAAATCCTCCATCAGC 1320
     TGCTCAGACGTGGAAAGGACGAGAGGGATTAAGTCACATACCTGAGAAAATTTTATTTTC 1380
     TGCCTATTTAGTCAATCATGGCCTGTATCTTAACGTTTTGATTTCAATATTTCTAAGTGT 1440
     CTGTGAGAAATACTTTTCGAAACCACAATAACAACAGATATTATCTTCTCAAGCGGGAGA 1500
     AACCCAATACCTGGTCTCATTCATTGGTGAACGTGGTTGCGCACGCCTGCATTTTCTTAA 1560
     TGTTACCACAAATTACAAATGTTCTCATTTTACAATTTTGAAAATGGACTTTCCTGTTAG 1620
     GTTTCTACGCTTCTGGTTCTTTATAGTGTTTTGAGTAGGCATGCTCTCCTGCAGACTGGG 1680
     TCCTAACACTAATCCAGTTGCTGCGGATCTCTGTAAGGTGTATTACCGATGCCCTCCACT 1740
     AGAGAGCACTGTAATGAAATCTCCGTGAACTTCAGTGTTCACCCACATAGTTCTCAGGAC 1800
     AGCCGCTGTAGTAGAATCTCCTAAGCCTTGCTGAGAAGTCAAGATTGTACTATGATTGCT 1860
     CCTAATGCTGAACTCAAAGAGGGAGTTTGATTTGCTGAGTTCAGCTGAACTCTTTGATTT 1920
     TTATACCCTAGTGTATTATGAACCTAGGTGGATGTTTAGTGACCAATCTGTTGGGGTCTT 1980
     TTGTATATAGTTGATAATTCTGCAAGAAGTTCAGTTTGTACAAATCTTCTGTGCACATGC 2040
     ATTTGTTACAAATAAAGAGCATTCTTTTTGAAAAAAAAAAAAAAAAAA             2088
```

Figure S1. Nucleotide and deduced amino acid sequences of mouse SULT7A1. Nucleotides are numbered in the 5' to 3' direction with the adenosine of the translation initiation codon designated as +1. The translation stop codon is indicated by an asterisk. SULT7A1 cDNA consists of a 5'-terminal untranslated sequence (5'-UTR) of 115 bp, an open reading frame (ORF) of 870 nucleotides encoding a 290 amino acid polypeptide, a 3'-UTR of 1745 bp with two typical polyadenylation signal sequence (AATAAA), and a poly(A) tail. Two amino acid residues, Ser3 and Gly263, are different from other reference sequences (GenBank accession numbers; BC151094, BC151096, and BC172155).

Figure S2

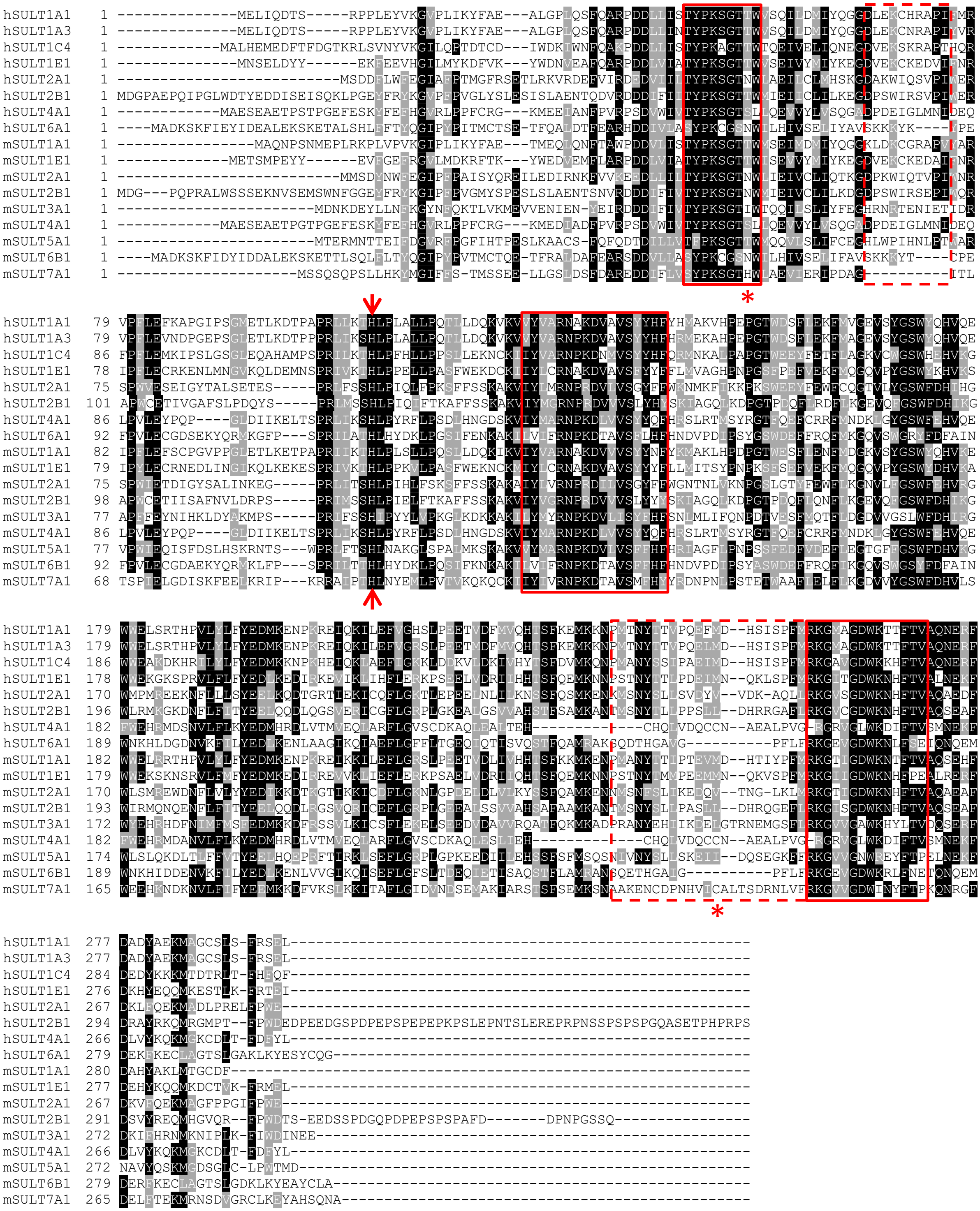

Figure S2. **Amino acid sequence alignment of human and mouse SULTs.** Identical amino acid residues are marked by grey background. Solid lines indicate the 'signature sequences' involved in the binding of PAPS. Arrows indicate the catalytic residue His conserved among all known SULTs. Asterisks indicate unique residues, His51 and Cys234, in the active site of SULT7A1 as shown in Fig. 4b.

Figure S3

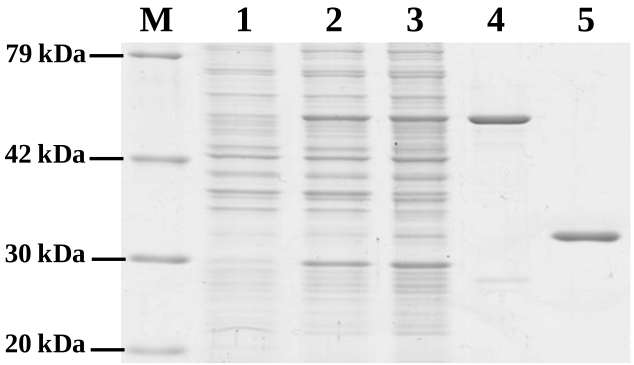

Figure S3. SDS-gel electrophoretic patterns of recombinant mouse SULT7A1. Samples were subjected to SDS-PAGE on a 12% gel, followed by Coomassie Blue staining. The purification procedure was described in Experimental procedures. Lane 1, before IPTG induction; lane 2, after IPTG induction; lane 3, cytosol portion; lane 4, GST-fusion protein; lane 5, purified enzyme.

Figure S4

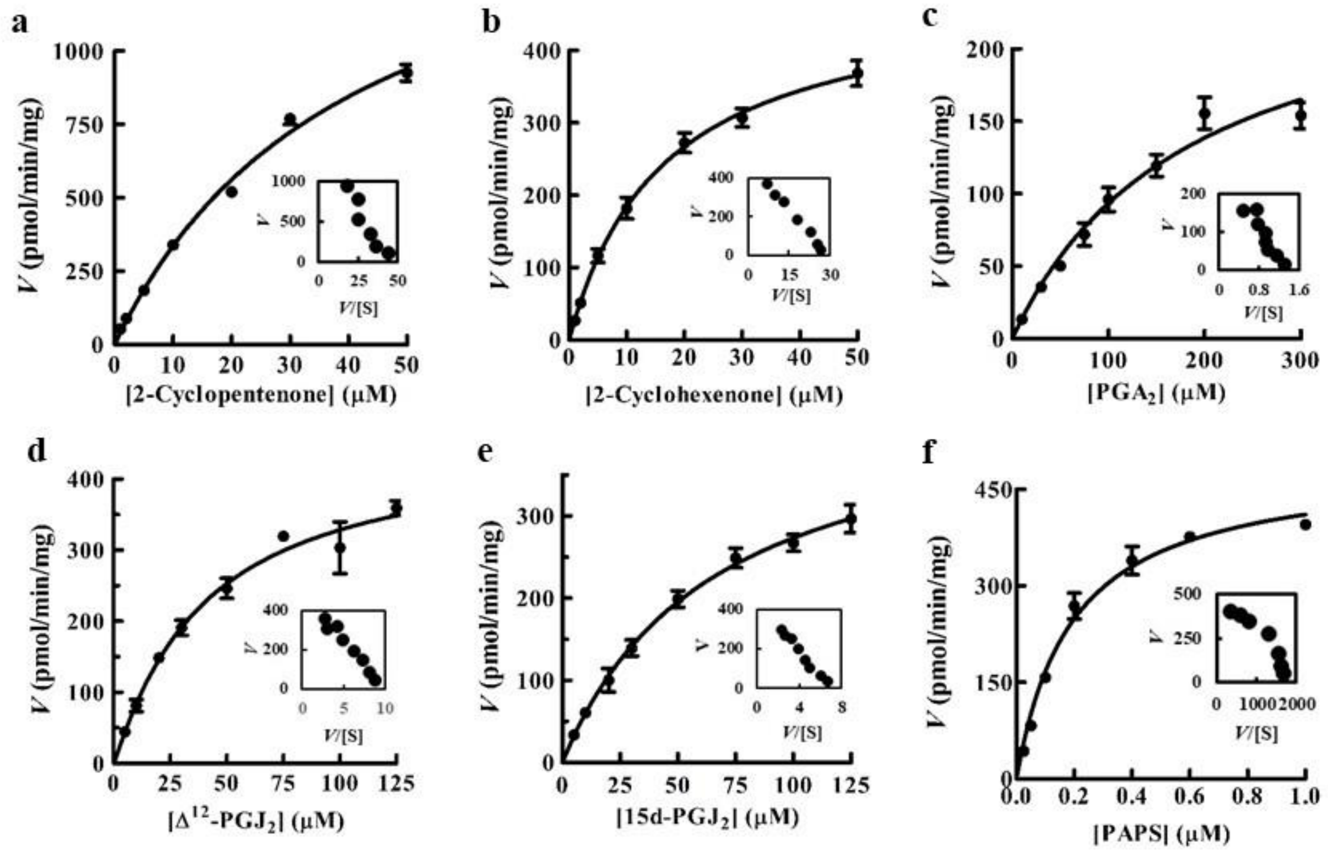

Figure S4. Kinetics analyses of the sulfonation catalyzed by SULT7A1. Kinetic assay was carried out to perform the enzymatic assays with the different concentrations of 2-cyclopentenone (a), 2-cyclohexanone (b), PGA_2 (c), delta12-PGJ_2 (d), 15d-PGJ_2 (e), or PAPS (f) under standard assay conditions as described in Materials and Methods. The fitting curves were generated using Michaelis-Menten kinetics. Eadie-Hofstee plots are inserted under each fitting curve. The data are calculated mean ± SD (n = 3).

Figure S5

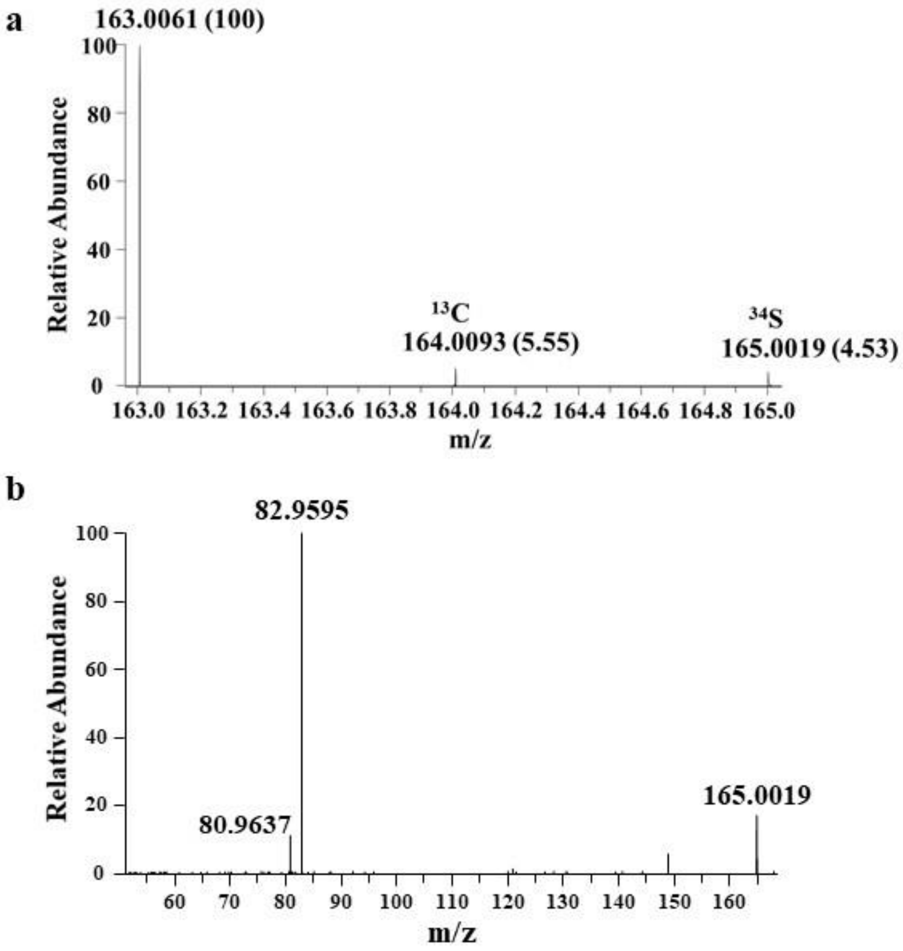

Figure S5. **Detailed MS analysis of the sulfonated product of 2-cyclopentenone. a**, Isotopic MS spectrum of the sulfonated product of 2-cyclopentenone. Intensity of each isotopic spectrum, ^{13}C (5.55%) or ^{34}S (4.53%), was expressed in relative values (%) against monoisotopic spectrum at m/z of 163.0061. **b**, MS/MS spectrum of ^{34}S-isotopic ion (165.0019 m/z).

Figure S6

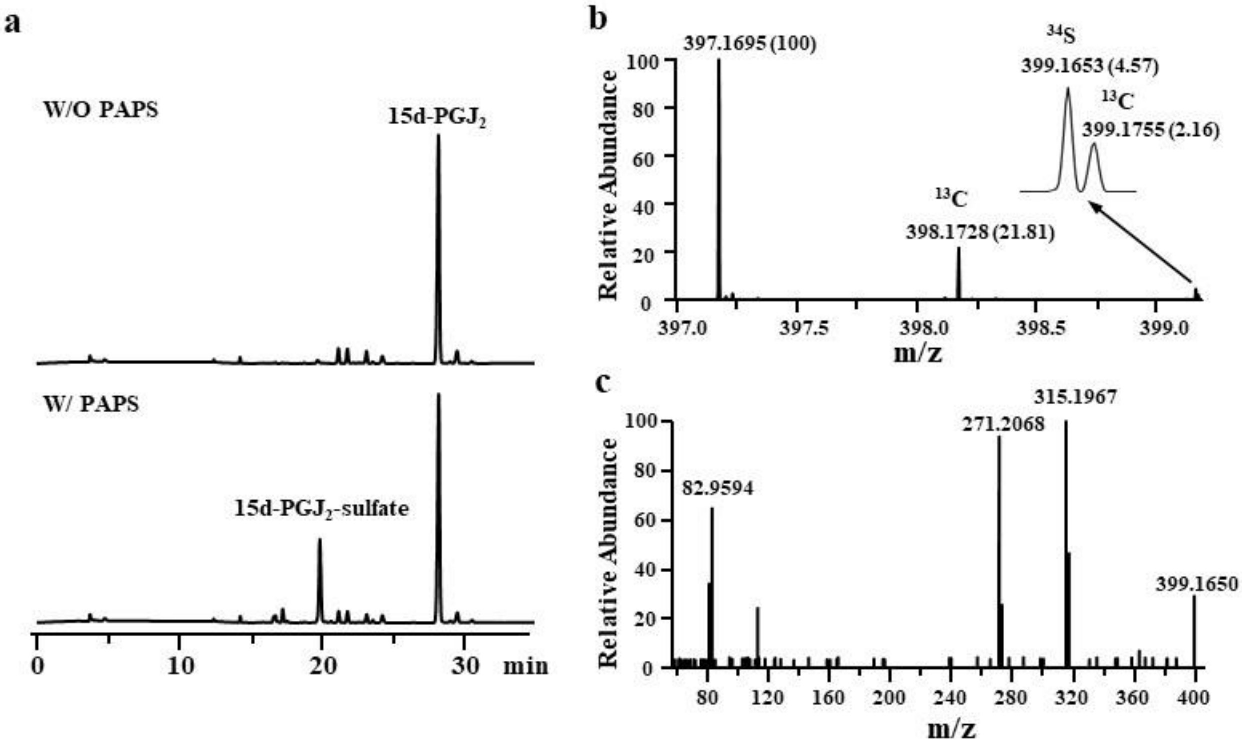

Figure S6. Chemical analyses of the sulfonated product of 15d-PGJ_2. **a**, HPLC analysis of sulfonation reaction mixture of 15d-PGJ_2. Upper and lower spectra show the reaction mixture without PAPS and with PAPS, respectively. **b**, Isotopic MS spectrum of the sulfonated product of 15d-PGJ_2. Intensity of each isotopic spectrum, ^{13}C (21.81%), ^{34}S (4.57%), or 2 x ^{13}C (2.16%) was expressed in relative values (%) against monoisotopic spectrum at m/z of 397.1695. **c**, MS/MS spectrum of ^{34}S-isotopic ion (399.1650 m/z).

Figure S7

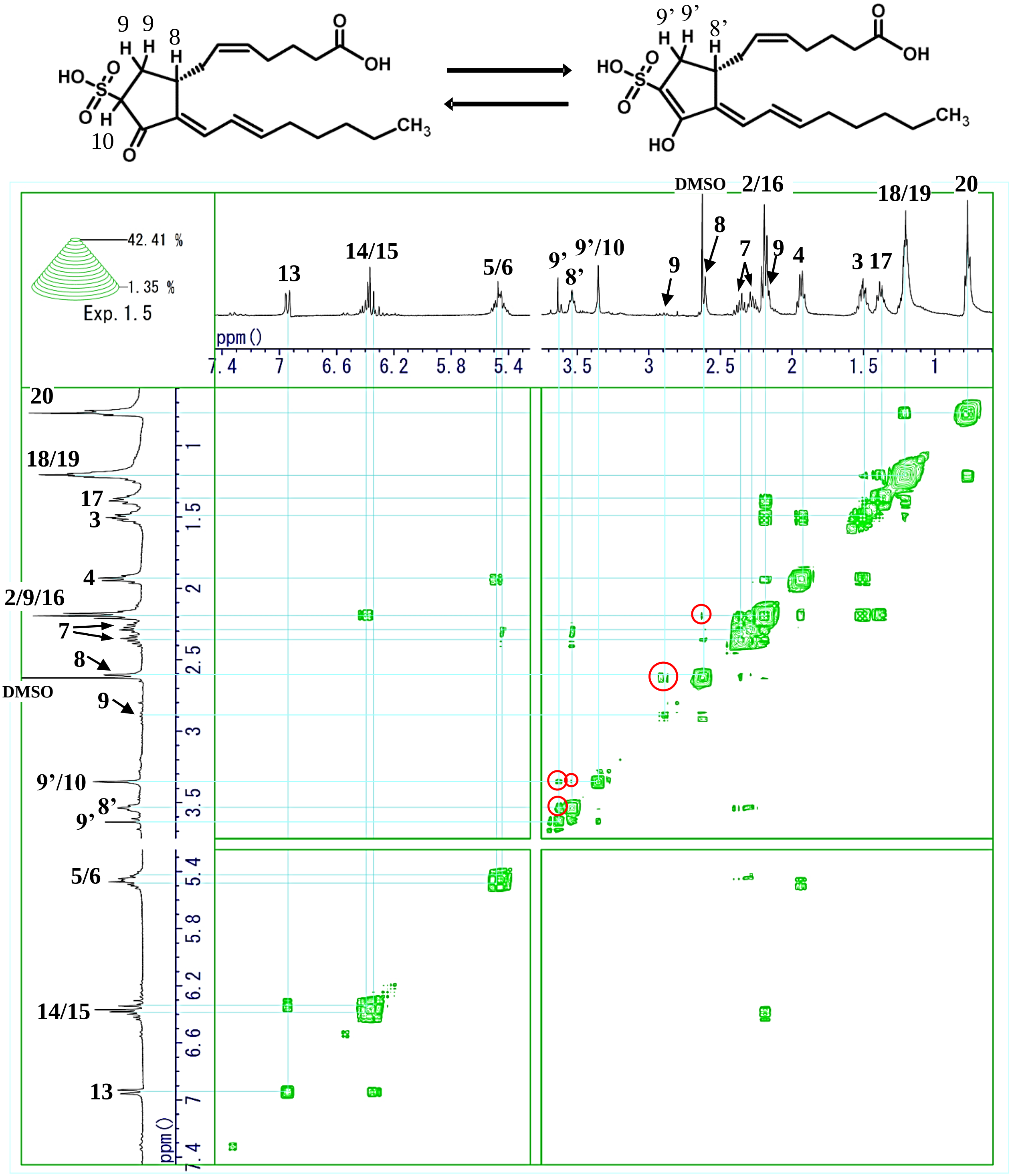

Figure S7. Correlation spectroscopy (COSY) analysis of the sulfonated product of 15d-PGJ$_2$. Each of protons numbered in chemical structures was assigned for the corresponding spectrum. Correlations among H8, H9, and H10 are represented by the line circles.

Figure S8

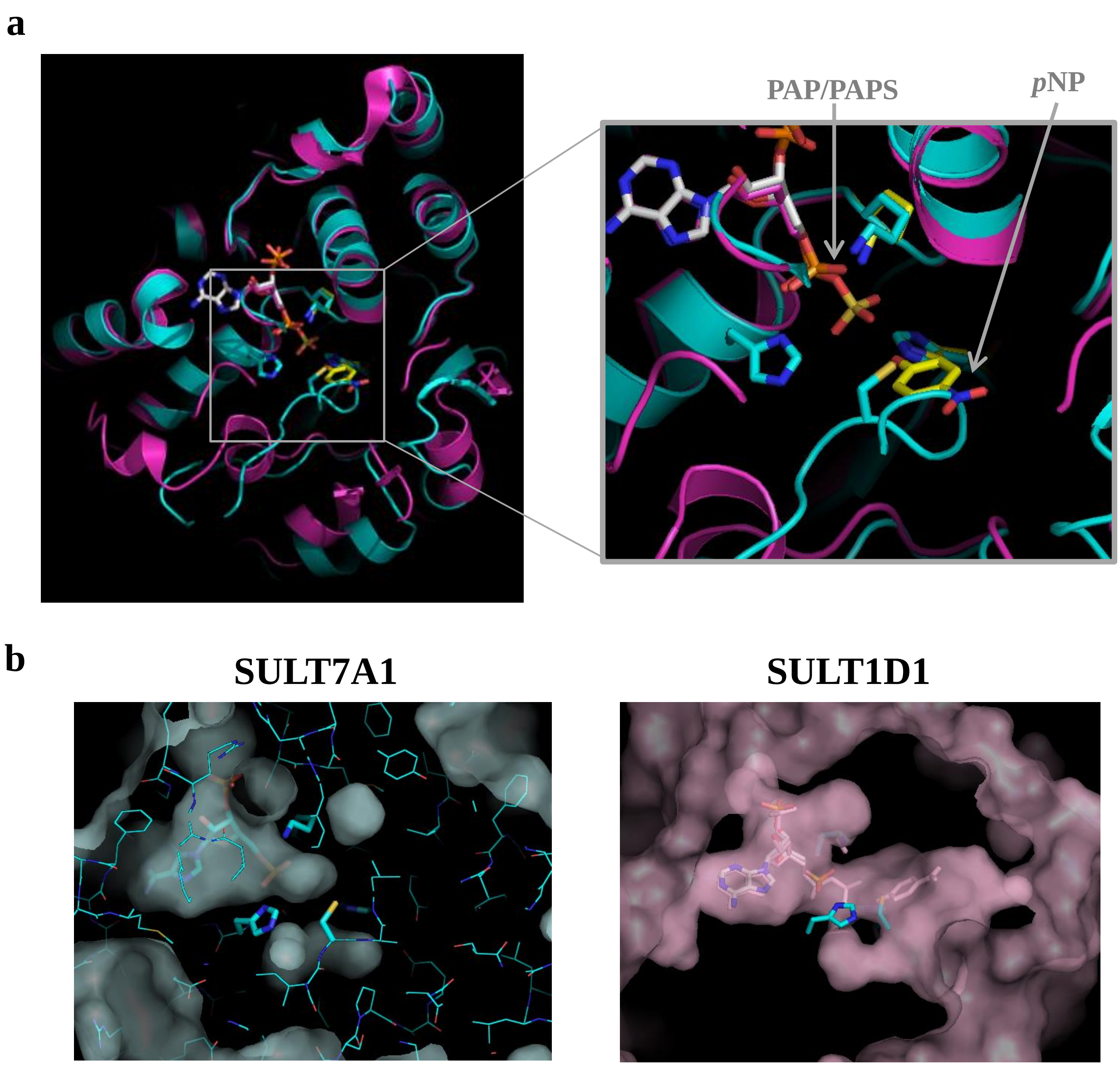

Figure S8. **Structural comparison of SULT7A1 with SULT1D1. a**, Ribbon diagram of the overall structure of SULT7A1, in complex with PAP, with SULT1D1, in complex with PAPS and *p*NP. Structure of SULT7A1 and SULT1D1 are colored slate blue and magenta, respectively. Close-up view of the active sites is shown in right panel. **b**, Substrate docking space comparison of SULT7A1 with SULT1D1.

Figure S9

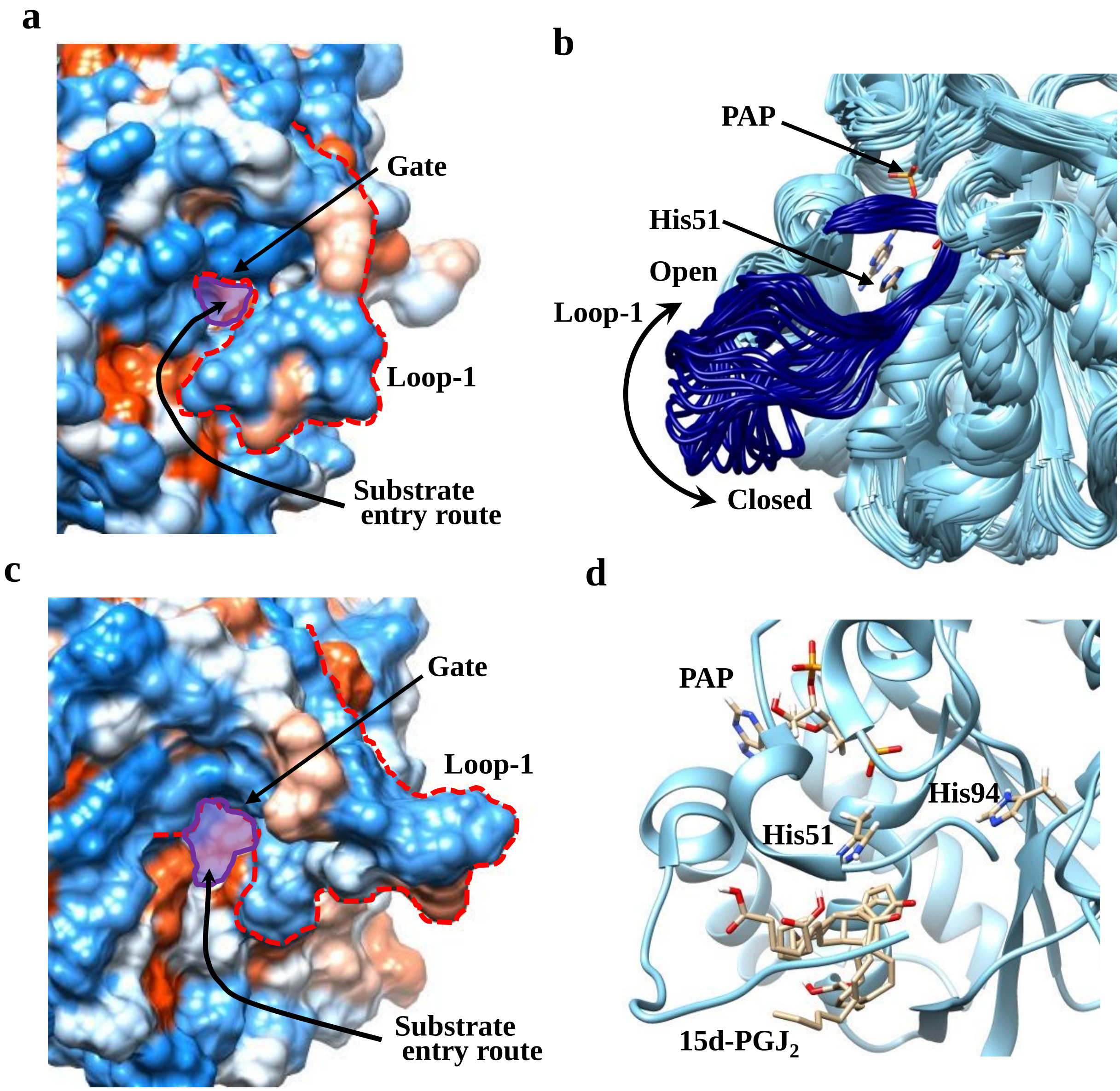

Supplementary Figure S9. **Simulation analysis of the flexible loop of SULT7A1 . a**, Hydrophobicity surface of SULT7A1 structure deleted Cys[234]. Loop-1 and substrate entry gate are shown by red and purple lines, respectively. Cavity to the substrate entry gate is shown as a substrate entry route. **b**, Snapshots of the simulated conformation of SULT7A1. Representative structures of each cluster out of 32 clusters were aligned. Loop-1 are colored dark blue. **c**, Hydrophobicity surface of SULT7A1 with opened loop-1 (**c**). The structure with opened loop-1 (c) was obtained from the results of molecular dynamics simulations. Loop-1 and substrate entry gate are shown by red and purple lines, respectively. Cavity to the substrate entry gate is shown as a substrate entry route. **d**, 15d-PGJ_2 docked into the active site of SULT7A1 with an opened loop-1 . Simulated superposition of 15d-PGJ_2 are colored by khaki.

Figure S10

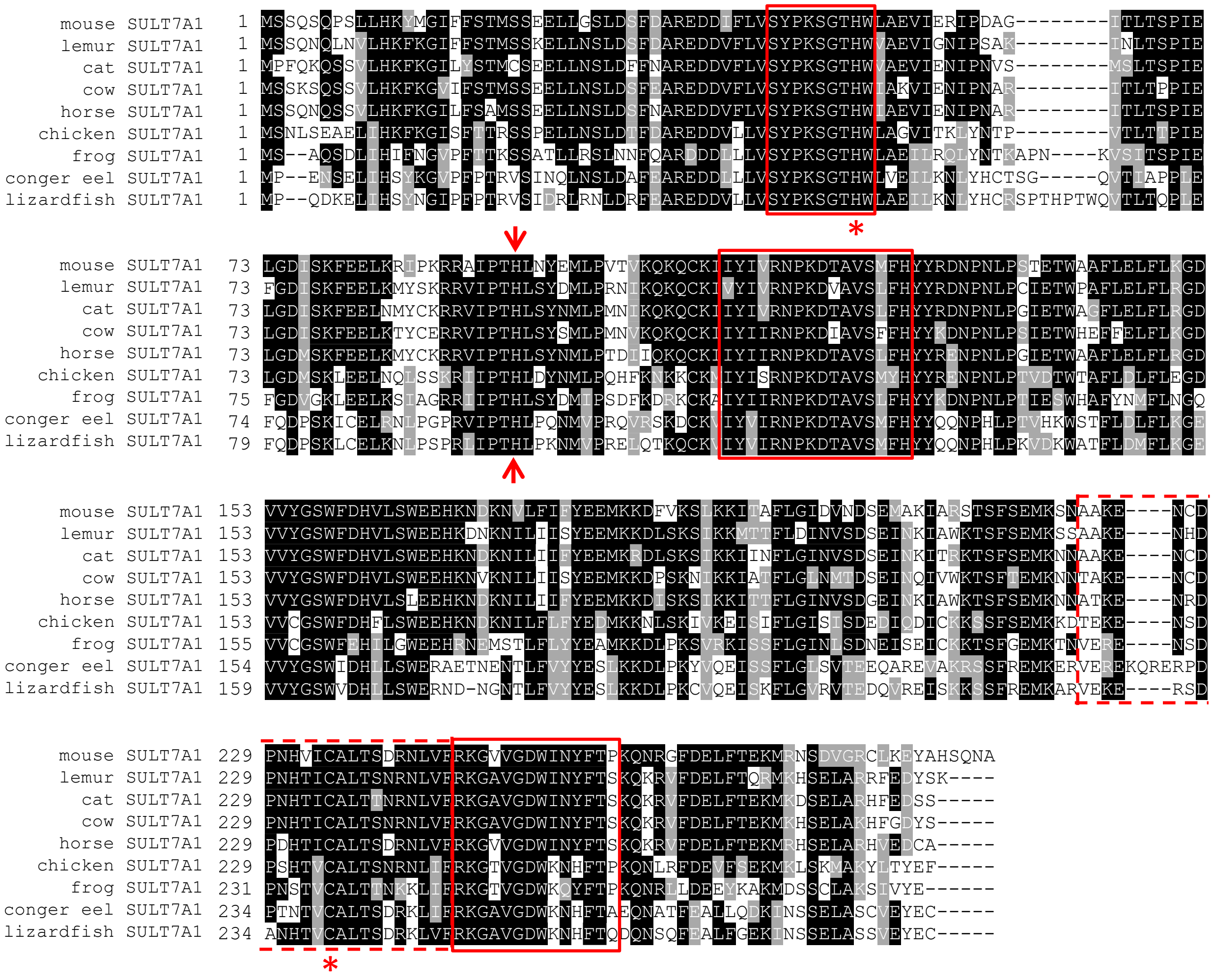

Figure S10. **Amino acid sequence alignment of SULT7A1.** Deduced amino acid sequences of putative lemur SULT7A1 (XP_012640945), cat SULT7A1 (XP_006933627), cow SULT7A1 (XP_024848365), horse SULT7A1 (XP_014596221), chicken SULT7A1 (XP_040516272), frog SULT7A1 (NP_001120086), conger eel SULT7A1 (XP_061085739), lizardfish SULT7A1 (KAJ8411219) were aligned. Identical amino acid residues are marked by grey background. Solid lines indicate the 'signature sequences' involved in the binding of PAPS. Arrows indicate the catalytic residue His conserved among all known SULTs. Asterisks indicate unique residues, His51 and Cys234, in the active site of SULT7A1 as shown in Fig. 4b.

Figure S11

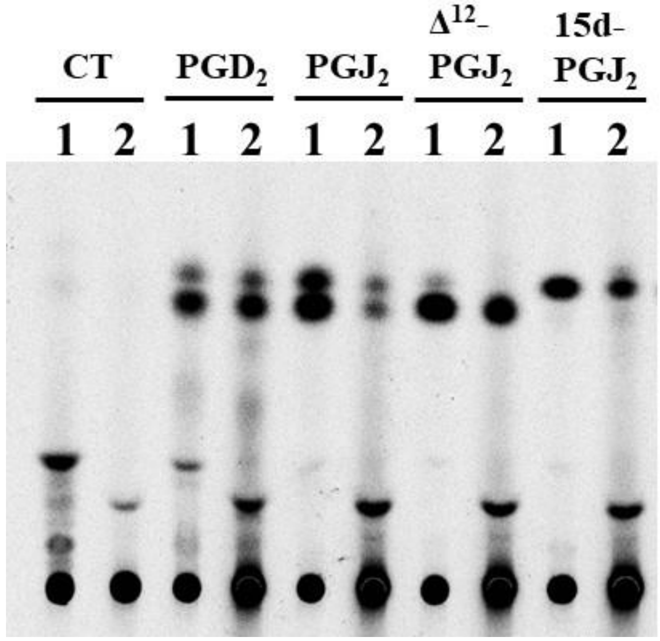

Figure S11. Analysis of [^{35}S]sulfonated products of prostaglandins. The figure shows the autoradiograph taken from the TLC plate. Lane 1 and 2 correspond to the reaction mixture of *in vitro* enzyme assay (lane 1) or labeling media of SULT7A1-expressing BHK-21 cells (lane 2). CT refers to the control sample without added prostaglandins.

Figure S12

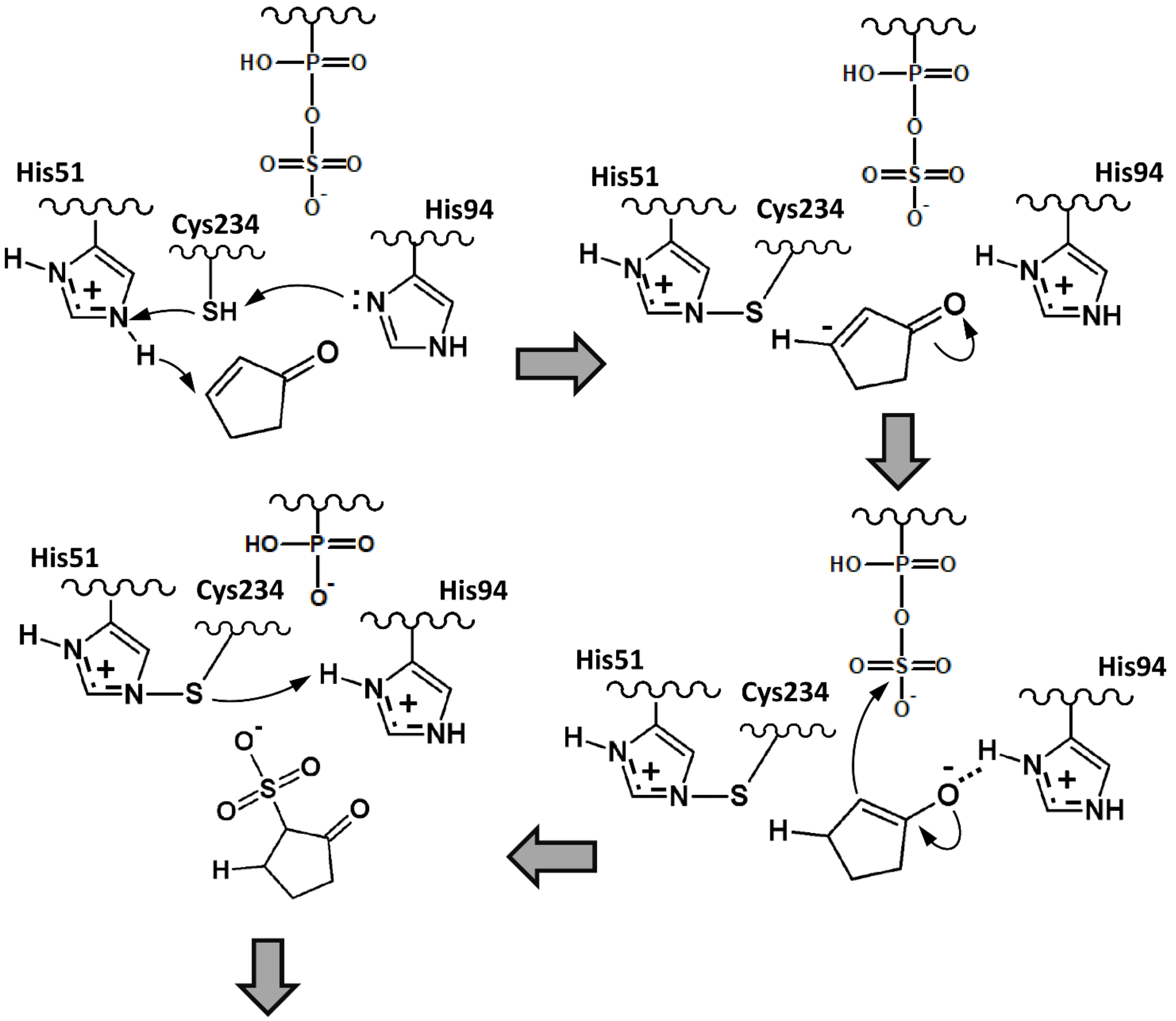

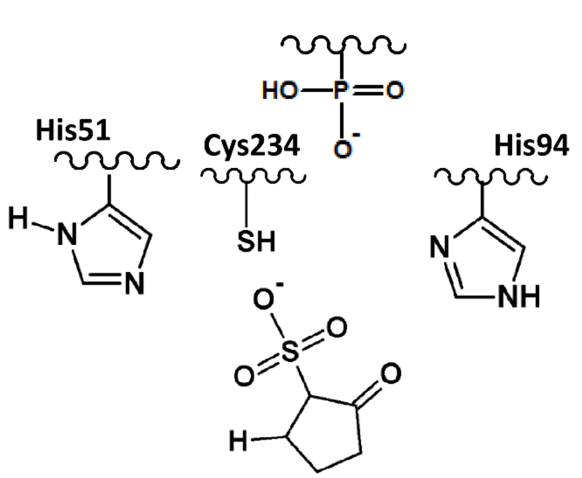

Figure S12. The proposed reaction mechanism of the unconventional sulfonation reaction by SULT7A1. Arrows in the catalytic residue-PAPS-substrate structure indicate the transition of electron pair.

Figure S13

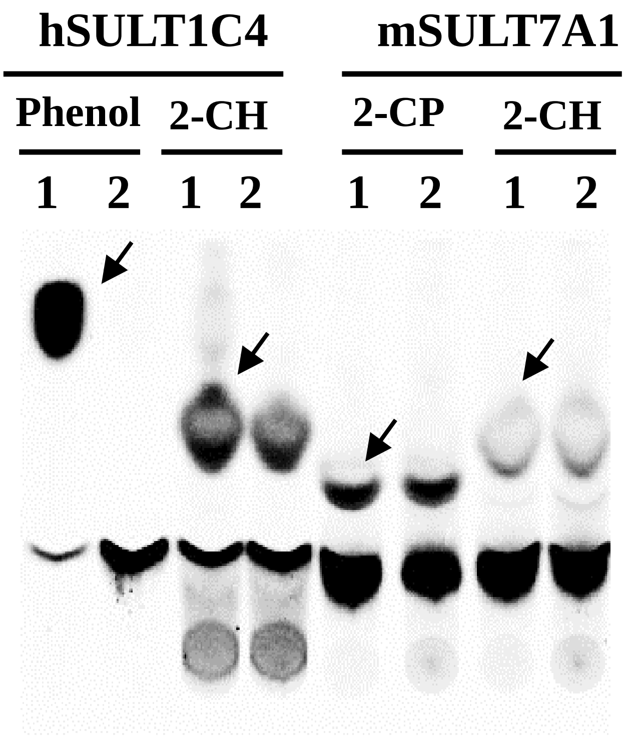

Figure S13. Sulfonation of α,β-unsaturated carbonyl compounds by human SULTs and sulfatase assay for [^{35}S]sulfonated products of α,β-unsaturated carbonyl compounds. (a) Specific sulfonating activity of human SULTs toward 2-cyclohexenone. The activity refers to pmol substrate sulfonated/min/mg purified enzyme. ND refers to activity not detected with the detection limit estimated to be 1.0 pmol/min/mg protein. Data represent mean ± SD derived from three determinations. (b) The figure shows the autoradiograph taken from the TLC plate. Lane 1 and 2 correspond to the reaction mixture without sulfatase (lane 1) or with (lane 2). 2-CH and 2-CP refers to the 2-cyclohexenone and 2-cyclopentenone used as the substrate, respectively. Arrows indicate the sulfonated derivatives.

Table S1

Table S1. **NMR data of 15d-PGJ$_2$ and the sulfonated product.**

	15d-PGJ$_2$-sulfate		15d-PGJ$_2$	
H	d (ppm)	J (Hz)	d (ppm)	J (Hz)
2	2.28 m	COSY[a]	2.26 m	COSY
3	1.59 m	COSY	1.61 quin	7.4
4	2.02 q	6.9	2.04 m	COSY
5	5.55 m	COSY	5.32 m	COSY
6	5.55 m	COSY	5.45 m	COSY
7	2.36 m, 2.45 m	COSY	2.44 m, 2.61 m	COSY
8	3.62 m	COSY	3.71 m	COSY
8'	2.71 m	COSY		
9	3.72 m, 3.44 m	COSY	7.66 dd	5.8, 2.5
9'	2.95 m, 2.28 m	COSY		
10	3.44 m		6.32 m	COSY
13	7.03 d	10.6	6.91 d	11.4
14	6.45 m	COSY	6.48 dd	11.6, 5.4
15	6.45 m	COSY	6.32 m	COSY
16	2.28 m	COSY	2.26 m	COSY
17	1.47 quin	6.9	1.50 quin	7.2
18	1.29 m	COSY	1.35 m	COSY
19	1.29 m	COSY	1.35 m	COSY
20	0.86 t	6.8	0.92 t	6.7

[a]COSY refers to the correlations observed in correlation spectroscopy (COSY) analysis and the coupling constants were not determined due to the multiple or overlapping proton signals.

Table S2

Table S2. Specific sulfonating activity of human SULTs toward 2-cyclohexenone.

	Specific activity (pmol/min/mg)	
	10 μM	100 μM
hSULT1A3	ND	2.34 ± 0.89
hSULT1B1	ND	10.44 ± 2.81
hSULT1C2	ND	2.46 ± 1.24
hSULT1C4	54.42 ± 1.46	529.8 ± 10.3

The activity refers to pmol substrate sulfonated/min/mg purified enzyme. ND refers to activity not detected with the detection limit estimated to be 1.0 pmol/min/mg protein. Data represent mean ± SD derived from three determinations.

Table S3

Table S3. **Lists of oligonucleotide primers.**

**I. For cloning of mouse SULT7A1 cDNA**		
**ORF cloning**	**Sense**	**5’-CGGGATCCATGTCATCCCAGAGTCAGCCAA-3’**
	**Antisense**	**5’-GGAATTCCTCACGCATTCTGCGAATGCGCA-3’**
**5’-RACE**	**RT antisense**	**5’-AAGGGAGATTGGGGTT-3’**
**1st PCR**	**Sense**	**5’-GGATACCTAAGAGAAGAGCT-3’**
	**Antisense**	**5’-TTTTGGGTAGGAAACCAGAA-3’**
**Nested PCR**	**Sense**	**5’-TATGAAATGCTTCCTGTGACTG-3’**
	**Antisense**	**5’-CTAGCATCAAAAGAGTCCA-3’**
**3’-RACE**	**Anchored oligo (dT)**	**5’-GGCCACGCGTCGACTAGTAC-(dT)$_{17}$-3’**
**1st PCR**	**Sense**	**5’-GGATACCTAAGAGAAGAGCT-3’**
	**Anchored antisense**	**5’-GGCCACGCGTCGACTAGTAC-3’**
**Nested PCR**	**Sense**	**5’-AAGATCGCTCGGAGTACGTC-3’**
	**Anchored antisense**	**5’-GGCCACGCGTCGACTAGTAC-3’**
**II. For site-directed mutagenesis**		
**H51A**	**Sense**	**5’-CAAAATCTGGCACTGCTTGGCTGGCAGAAG-3’**
	**Antisense**	**5’-CTTCTGCCAGCCAAGCAGTGCCAGATTTTG-3’**
**H94A**	**Sense**	**5’-GAGCTATCCCGACAGCTCTGAACTATGAAA-3’**
	**Antisense**	**5’-TTTCATAGTTCAGAGCTGTCGGGATAGCTC-3’**
**C234A**	**Sense**	**5’-CCAATCACGTCATCGCTGCCCTCACGTCCG-3’**
	**Antisense**	**5’-CGGACGTGAGGGCAGCGATGACGTGATTGG-3’**
**III. For RT-PCR analysis**		
**mSULT7A1**	**Sense**	**5’-GGATACCTAAGAGAAGAGCT-3’**
	**Antisense**	**5’-AAGGGAGATTGGGGTTGTCC-3’**
**β-actin**	**Sense**	**5’-ACAGGATGCAGAAGGAGATCAC-3’**
	**Antisense**	**5’-TCATACTCCTGCTTGCTGATCC-3’**
**IV. For cloning of prostanoid receptors cDNA**		
**DP**	**Sense**	**5’-CCGGAATTCATGAAGTCGCCGTTCTACCGC-3’**
	**Antisense**	**5’-GCTCTAGATCACAGACTGGATTCCATGTTA-3’**
**EP2**	**Sense**	**5’-CCGGAATTCATGGGCAATGCCTCCAATGAC-3’**
	**Antisense**	**5’-GCTCTAGATCAAAGGTCAGCCTGTTTACTG-3’**
**EP4**	**Sense**	**5’-CCGGAATTCATGTCCACTCCCGGGGTCAAT-3’**
	**Antisense**	**5’-GCTCTAGATTATATACATTTTTCTGATAAG-3’**
**IP**	**Sense**	**5’-CCGGAATTCATGGCGGATTCGTGCAGGAAC-3’**
	**Antisense**	**5’-GCTCTAGATCAGCAGAGGGAGCAGGCGACG-3’**

Table S4

Table S4. Data collection and refinement statistics.

Data collection	
Space group	$P2_1$
Unit cell parameters	a = 166.17 Å
	b = 81.13 Å
	c = 166.18 Å
	β = 119.9°
Wavelength (Å)	1.0
Resolution range (Å)	50.0 - 2.08
No. of reflections	
Observed / Unique	852,959 / 229,447
Redundancy	3.7 (3.5)
R_{sym}[a,b]	0.085 (0.912)
$I/\sigma(I)$[a]	16.8 (1.5)
Completeness (%)	99.8 (98.8)
Refinement statistics	
Resolution range (Å)	50.0 - 2.08
No. of reflections	
Working set / Test set	217,914 / 11,949
Completeness (%)	99.7
R_{cryst}[c] (%) / R_{free}[d] (%)	22.1 / 23.6
Root mean square deviation	
Bond length (Å)	0.020
Bond angles (°)	1.9
Average B-factor (Å^2) / No. of atoms	
Protein	42.3 / 1,149,347
Water	42.4 / 1679
PAP	42.7 / 324
Ramachandran analysis	
Favored (%)	87.9
Allowed (%)	9.9
Outlier (%)	2.2

[a]Values in parentheses are for the highest-resolution shell.
[b]$R_{sym} = \sum(I - \langle I \rangle)/\sum\langle I \rangle$, where I is the intensity measurement for a given refraction and $\langle I \rangle$ is the average intensity for multiple measurements of this refraction.
[c]$R_{cryst} = \sum|F_{obs} - F_{cal}| \sum F_{obs}$, where F_{obs} and F_{cal} are observed and calculated structure factor amplitudes.
[d]R_{free} value was calculated for R_{cryst}, using only an unrefined randomly chosen subset of reflection data (5%).
